# Activation of Intestinal HIF2α Ameliorates Iron‐Refractory Anemia

**DOI:** 10.1002/advs.202307022

**Published:** 2024-01-20

**Authors:** Yingying Yu, Yunxing Su, Sisi Yang, Yutong Liu, Zhiting Lin, Nupur K. Das, Qian Wu, Jiahui Zhou, Shumin Sun, Xiaopeng Li, Wuyang Yue, Yatrik M. Shah, Junxia Min, Fudi Wang

**Affiliations:** ^1^ The First Affiliated Hospital The Second Affiliated Hospital Institute of Translational Medicine School of Public Health State Key Laboratory of Experimental Hematology Zhejiang University School of Medicine Hangzhou 310058 China; ^2^ The First Affiliated Hospital Basic Medical Sciences School of Public Health Hengyang Medical School University of South China Hengyang 421001 China; ^3^ Internal Medicine Division of Gastroenterology University of Michigan Ann Arbor MI 48109 USA; ^4^ Department of Molecular & Integrative Physiology University of Michigan Ann Arbor MI 48109 USA; ^5^ International Institutes of Medicine Zhejiang University School of Medicine Yiwu Zhejiang China

**Keywords:** anemia of inflammation, chemotherapy‐induced anemia, FG‐4592, HIF, hypoxia, iron‐refractory iron deficiency anemia

## Abstract

In clinics, hepcidin levels are elevated in various anemia‐related conditions, particularly in iron‐refractory anemia and in high inflammatory states that suppress iron absorption, which remains an urgent unmet medical need. To identify effective treatment options for various types of iron‐refractory anemia, the potential effect of hypoxia and pharmacologically‐mimetic drug FG‐4592 (Roxadustat) are evaluated, a hypoxia‐inducible factor (HIF)‐prolyl hydroxylase (PHD) inhibitor, on mouse models of iron‐refractory iron‐deficiency anemia (IRIDA), anemia of inflammation and 5‐fluorouracil‐induced chemotherapy‐related anemia. The potent protective effects of both hypoxia and FG‐4592 on IRIDA as well as other 2 tested mouse cohorts are found. Mechanistically, it is demonstrated that hypoxia or FG‐4592 could stabilize duodenal Hif2α, leading to the activation of *Fpn* transcription regardless of hepcidin levels, which in turn results in increased intestinal iron absorption and the amelioration of hepcidin‐activated anemias. Moreover, duodenal *Hif2α* overexpression fully rescues phenotypes of *Tmprss6* knockout mice, and *Hif2α* knockout in the gut significantly delays the recovery from 5‐fluorouracil‐induced anemia, which can not be rescued by FG‐4592 treatment. Taken together, the findings of this study provide compelling evidence that targeting intestinal hypoxia‐related pathways can serve as a potential therapeutic strategy for treating a broad spectrum of anemia, especially iron refractory anemia.

## Graphical abstract

In this article, it is demonstrated that targeting the duodenal Hif2α‐Fpn axis as a novel strategy to improve refractory hepcidin‐activated anemias, including iron‐refractory iron‐deficiency anemia (IRIDA), inflammatory anemia and chemotherapy‐induced anemia, in mice, which provides compelling evidence for further clinical translation.

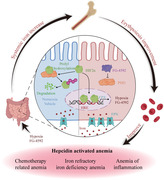

## 1 Introduction

Nearly a quarter of the world's population suffers from anemia.^[^ ^1^, ^2^ ^]^ Anemia of inflammation (AI) and iron‐deficiency‐induced anemia are the two most common forms worldwide. And they often coexist, causing a severe additional burden on the recovery from their primary underlying diseases in people with a high prevalence of nutritional deficiencies, chronic infections, or chronic systemic inflammation.^[^ ^3^, ^4^ ^]^

Mechanisms causing iron deficiency with inflammation are centered on increased hepatic hepcidin (encoded by *HAMP*) and decreased iron exporter ferroportin (FPN, also known as SLC40A1). This dynamic interplay hinders iron absorption from duodenal enterocytes and iron mobilization by the reticuloendothelial system, posing a significant challenge to effective oral iron supplementation. Historically, heightened iron levels or inflammation could upregulate hepcidin expression, whereas erythropoiesis or hypoxia inhibits hepcidin expression.^[^ ^5^, ^6^, ^7^ ^]^ When serum transferrin is saturated, the homeostatic iron regulator (HFE) is displaced from transferrin receptor‐1 (TFR1) to form a complex with TFR2 and hemojuvelin (HJV). This complex activates the bone morphogenetic proteins (BMP)/small mothers against decapentaplegic (SMAD) signaling cascade, ultimately causing upregulated hepcidin expression. However, this canonical hepcidin regulatory pathway is counteracted by the action of matriptase 2 (encoded by *TMPRSS6*),^[^ ^8^ ^]^ a serine protease that cleaves and generates a soluble form of HJV.^[^ ^9^ ^]^ Loss‐of‐function mutations in *TMPRSS6* induce iron‐refractory iron deficiency anemia (IRIDA).^[^ ^10^, ^11^, ^12^ ^]^ Previous studies have reported an association between *TMPRSS6* variants and an increased risk of iron deficiency.^[^ ^13^, ^14^ ^]^ Moreover, during active inflammation, interleukin 6 (IL‐6) activates Janus kinases (JAK)/signal transducer and activator of transcription 3 (STAT3), causing increased *Hamp* transcription. Conversely, upon anemia or hypoxia, kidney‐derived erythropoietin (EPO) increases in response to upregulated hypoxia‐inducible factor (HIF) 2α. Consequently, erythroblasts‐secreted erythroferrone (ERFE)^[^ ^6^, ^15^ ^]^ suppresses hepcidin production to maintain adequate iron absorption and normal erythropoiesis.^[^ ^16^, ^17^, ^18^, ^19^ ^]^

Recent studies have highlighted Hif2α as a critical adaptive transcription factor to regulate intestinal iron absorption, which is highly upregulated in response to increased systemic iron demand.^[^ ^20^, ^21^, ^22^ ^]^ Additionally, hepcidin inhibition leads to Hif2α activation, facilitating an appropriate iron absorptive response.^[^ ^23^ ^]^ However, it remains unclear whether Hif2α activation can effectively alleviate hepcidin‐activated anemias. Interestingly, it is observed highland residents and mountaineers had increased stability of HIFs and HIF‐targeted genes, including *EPO*, which in turn stimulated erythropoiesis.^[^ ^24^, ^25^, ^26^, ^27^ ^]^ However, it remains unclear whether hypoxia affects hepcidin‐activated and iron‐refractory anemias.

To address this clinically relevant question, we examined and demonstrated either hypoxia exposure or treatment with FG‐4592, an oral HIF‐prolyl hydroxylase (PHD) inhibitor, significantly accelerated the recovery from various forms of hepcidin‐activated anemias. Mechanistically, this benefit resulted from stabilized Hif2α and upregulated duodenal *Fpn* expression that facilitated increased duodenal iron absorption independent of hepatic *Hamp* expression. These findings suggest duodenal Hif2α‐Fpn axis could serve as a promising therapeutic target to treat anemias.

## 2 Results

### 2.1 Hypoxia Promotes Recovery from Iron‐Refractory Iron Deficiency Anemia (IRIDA)

**Figure 1:**
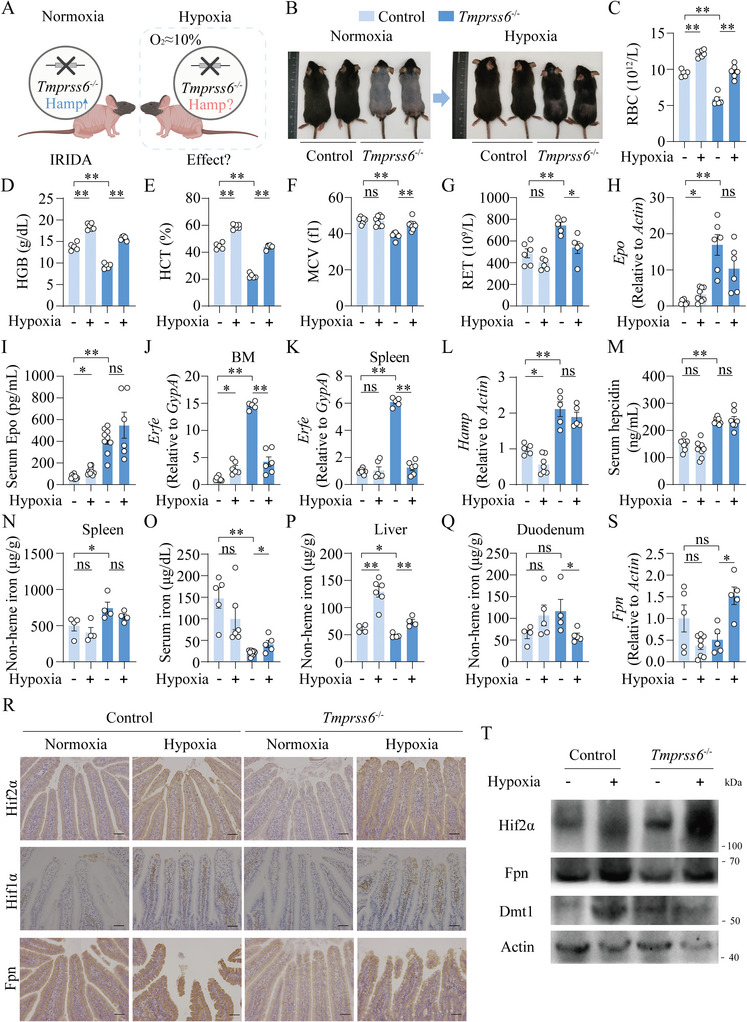
Global *Tmprss6* knockout mice display improved phenotypes of IRIDA upon hypoxia exposure for 4 weeks. A) A schematic diagram illustrates the key scientific question of the study, which is to investigate the potential effect of hypoxia on a mouse model of hepcidin‐activated anemia. B) Representative images of 8‐week‐old control and *Tmprss6* ^−/−^ mice before and after placement in a hypoxia chamber (O_2_:10%) for 4 weeks. C‐Q) RBC C), HGB D) HCT E), MCV F), RET G), renal *Epo* mRNA H), serum Epo I), *Erfe* mRNA normalized to the erythroid marker *GypA* in the bone marrow J) and spleen K), hepatic *Hamp* mRNA L), serum hepcidin M), splenic iron N), serum iron O), hepatic iron P) and duodenal iron Q) levels were detected in normoxia‐ and hypoxia‐exposed control and *Tmprss6* ^−/−^ mice. R–T) Representative duodenum images of Hif2α, Hif1α and Fpn immunohistochemistry R), duodenal *Fpn* mRNA S), representative western blot results of duodenal Hif2α, Fpn and Dmt1 T) were shown in normoxia‐ and hypoxia‐exposed control and *Tmprss6* ^−/−^ mice. Scale bars, 100 µm. Two‐way ANOVA with Tukey's post hoc test (for multi‐group comparisons), *n* = 4‐9 mice per group.

To examine the effect of hypoxia on IRIDA (**Figure** **1**A), we generated global *Tmprss6* knockout mice (*Tmprss6^−/−^*) (Figure S1A,B, Supporting Information), characterized by almost complete alopecia, which could be rescued by parenteral iron supplementation.^[^ ^10^, ^12^ ^]^ Mice exposed to hypoxia (10% O_2_) for 4 weeks demonstrated a near complete hair recovery (Figure 1B). In addition, hypoxia normalized the levels of red blood cells (RBCs), hemoglobin (HGB), hematocrit (HCT), mean corpuscular volume (MCV), and reticulocyte counts (RET) in *Tmprss6^−/−^* mice (Figure 1C–G). To examine the role of hypoxia in the development of proerythroblasts into mature RBCs in both bone marrow (BM) and spleen, we performed flow cytometry by gating the CD45 negative populations and using Ter119 and CD44 to analyze the erythroid lineage.^[^ ^16^ ^]^ In the BM, the cell counts of proerythroblasts (R1) were unchanged, but basophilic erythroblasts (R2), polychromatophilic erythroblasts (R3), orthochromatophilic erythroblasts (R4) decreased significantly, and mature erythrocytes (R5) significantly increased in *Tmprss6^−/−^* mice upon hypoxia treatment, suggesting medullary hematopoiesis was improved in *Tmprss6^−/−^* mice. Consequently, extramedullary hematopoiesis stress was significantly ameliorated as evidenced by the decreased counts of R2‐R4 and increased R5 in *Tmprss6* ^−/−^ mice upon hypoxia treatment (Figure S1C–G, Supporting Information), consistent with the improved spleen/body weight ratio and spleen histological assessments (Figure S1H,I, Supporting Information). Additionally, we found that control mice exhibited higher levels of renal *Epo*, serum Epo (Figure 1H,I), *Erfe* expression in BM (Figure 1J), and lower *Hamp* expression (Figure 1L), reflecting the typical erythropoiesis responses and iron demand upon hypoxia. Conversely, hypoxia exposure didn't affect renal *Epo*, serum Epo, hepatic *Hamp*, or serum hepcidin levels, but decreased *Erfe* levels in *Tmprss6^−/−^* mice (Figure 1H–M). Consistently, splenic non‐heme iron levels remain unchanged between normoxia and hypoxia in control and *Tmprss6^−/−^* mice (Figure 1N). However, serum iron levels were significantly upregulated in *Tmprss6^−/−^* mice upon hypoxia treatment, consistent with erythropoiesis recovery. (Figure 1O). Furthermore, hepatic non‐heme iron levels were normalized in hypoxia‐treated‐*Tmprss6^−/−^* mice (Figure 1P). Interestingly, hypoxia treatment decreased duodenal iron retention in *Tmprss6^−/−^* mice (Figure 1Q).

Next, we found the staining of both Hif2α and Hif1α in the duodenum, two well‐known master transcription factors that regulate cellular responses to hypoxia, are significantly stronger in hypoxia‐treated mice compared to normoxia‐exposed mice. And Hif2α appears to be present in the villi but Hif1α in the lamina propria (Figure 1R). Duodenal nuclear receptor coactivator 4 (Ncoa4),^[^ ^28^ ^]^ Fpn,^[^ ^29^ ^]^ and divalent metal transporter 1 (Dmt1)^[^ ^21^ ^]^ were reported previously as targets of HIFs and responsible for iron transport. Hypoxia treatment failed to trigger *Ncoa4* expressions among control and *Tmprss6^−/−^* mice (Figure S1J, Supporting Information), suggesting Ncoa4‐mediated intestinal ferritinophagy could not be required for erythropoiesis under hypoxia treatment. Duodenal *Dmt1* levels were unchanged (Figure S1K, Supporting Information), but *Fpn* levels were increased significantly in *Tmprss6^−/−^* mice upon hypoxia treatment (Figure 1S). Consistently, although hepcidin levels in hypoxia‐treated‐*Tmprss6^−/−^* mice remained unchanged, duodenal Fpn protein levels were significantly higher compared to normoxia‐treated‐*Tmprss6^−/−^* mice (Figure 1R–T; Figure S1L, Supporting Information). All these observations suggest hypoxia promotes iron absorption and erythropoiesis recovery of IRIDA by a regulatory mechanism independent of the canonical hepatic *Hamp*‐duodenum Fpn axis.

To further confirm whether hypoxia treatment could improve IRIDA through hepatic *Hamp*‐duodenum Fpn axis, we constructed a hepatocyte‐specific *Tmprss6* knockout mouse, *Tmprss6^flox/flox^; Alb‐Cre* (*Tmprss6*‐LKO). Decreased hepatic *Tmprss6* and increased hepatic *Hamp* expressions were confirmed by RT‐qPCR (Figure S2A,N, Supporting Information). Similar to *Tmprss6^−/−^* mice, *Tmprss6*‐LKO mice exposed to hypoxia for 4 weeks exhibited significantly improved hematological alterations, unchanged hepatic *Hamp* and serum hepcidin levels, but significantly higher serum iron levels, less duodenum iron retention with higher duodenal *Fpn* expressions (Figure S2, Supporting Information) compared to normoxia‐treated‐*Tmprss6*‐LKO mice. Together, these data suggest that hypoxia‐induced recovery of anemic phenotypes in these IRIDA mice mainly results from increased duodenal Fpn expression.

### 2.2 FG‐4592 Therapeutically Ameliorates IRIDA in Mice

**Figure 2:**
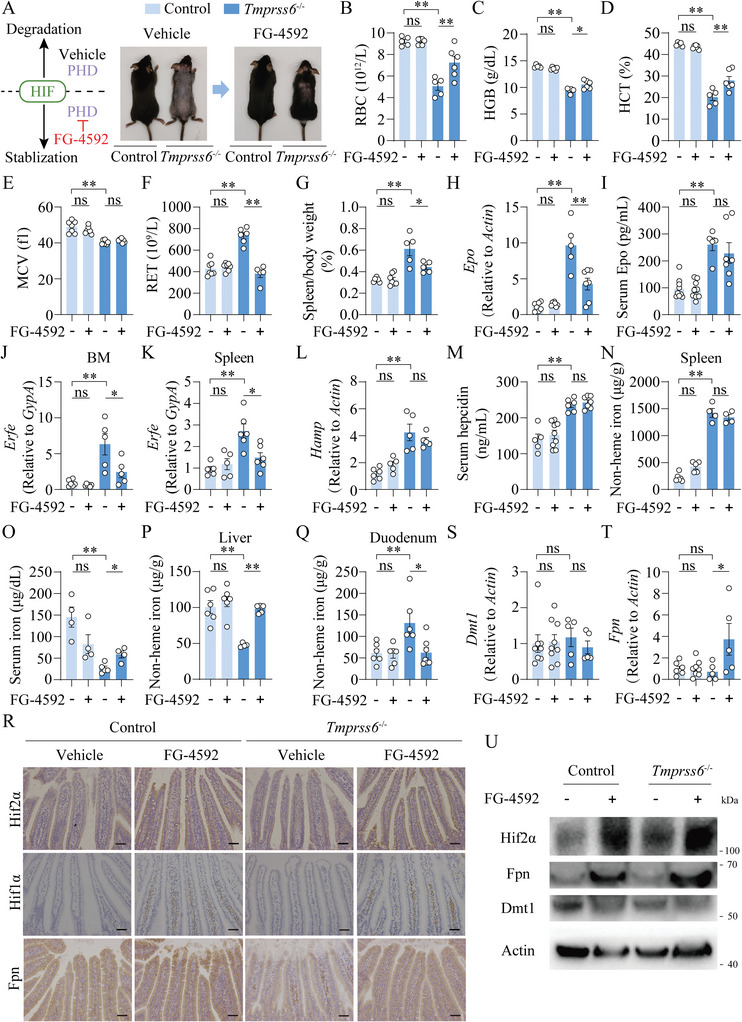
FG‐4592 improves phenotypes of IRIDA in global *Tmprss6* knockout mice. A) A schematic diagram illustrating the action mode of FG‐4592 (left) and representative images of *Tmprss6* ^−/−^ and their littermate control mice with or without FG‐4592 (30 mg kg^−1^ gavage, once a day) for 4 weeks (right). B‐Q) RBC B), HGB C), HCT D), MCV E) and RET F), spleen/body weight (%) G), renal *Epo* mRNA H), serum Epo I), *Erfe* mRNA normalized to the erythroid marker *GypA* in the bone marrow J) and spleen K), hepatic *Hamp* mRNA L), serum hepcidin M), splenic iron N), serum iron O), hepatic iron P) and duodenal iron levels Q) were detected in saline vehicle‐ and FG‐4592‐treated control and *Tmprss6* ^−/−^ mice. R–U) Representative duodenum images of immunohistochemistry for Hif2α, Hif1α and Fpn R), duodenal *Dmt1* S) and *Fpn* T) mRNA levels, and representative western blot results of Hif2α, Fpn and Dmt1 U) were shown in vehicle‐ and FG‐4592‐treated control and *Tmprss6* ^−/−^ mice. Scale bars, 100 µm. Two‐way ANOVA with Tukey's post hoc test (for multi‐group comparisons), *n* = 4–9 mice per group.

FG‐4592 is an orally reversible inhibitor of HIF‐PHD, which stabilizes HIFs and mimics natural hypoxia responses.^[^ ^30^, ^31^ ^]^ Therefore, we examined the effect of FG‐4592 in the IRIDA mouse models. After 4 weeks treatment of oral FG‐4592, *Tmprss6^−/−^* mice showed near complete hair growth and improved RBCs, HGB, HCT, and RET levels (**Figure** **2**A–F), and improved extramedullary hematopoiesis stress shown by decreased spleen mass and spleen H&E staining (Figure 2G; Figure S3, Supporting Information). Additionally, FG‐4592 decreased the levels of renal *Epo* and *Erfe* in BM and spleen, but didn't affect serum Epo, hepatic *Hamp*, serum hepcidin, or splenic non‐heme iron levels (Figure 2H–N) in *Tmprss6^−/‐^* mice. FG‐4592‐treated‐*Tmprss6^−/−^* mice exhibited elevated serum iron and hepatic non‐heme iron levels, decreased duodenal iron levels (Figure 2O–Q), increased duodenal protein expressions of Hif2α and Hif1α, unchanged *Dmt1* levels and increased *Fpn* mRNA and protein levels by IHC and western blot (Figure 2R–U) compared to vehicle‐treated‐*Tmprss6^−/−^* mice.

Similarly, FG‐4592‐fed *Tmprss6*‐LKO mice presented significant improvement in anemia‐ and iron‐related parameters without the change of hepatic *Hamp* levels compared to vehicle‐treated‐*Tmprss6*‐LKO mice (Figure S4, Supporting Information). Taken together, these data indicate FG‐4592 has potent activity for improving IRIDA independent of the hepatic *Hamp*‐duodenal Fpn axis.

### 2.3 FG‐4592 Improves Anemia of Chronic Kidney Disease via Upregulation of Duodenal *Fpn* through a Hepcidin‐Independent Pathway

**Figure 3:**
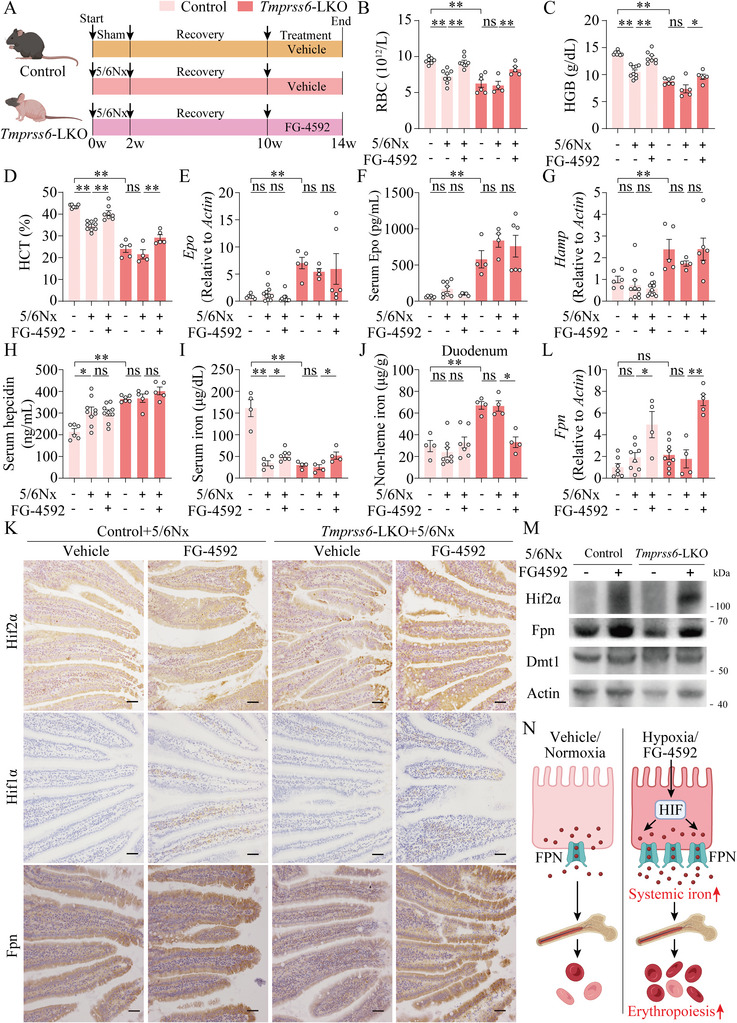
FG‐4592 ameliorates anemia of CKD in both control and *Tmprss6*‐LKO mice. A) A schematic diagram illustrats the experimental design of sham‐operated or 5/6 nephrectomy (5/6Nx)‐induced anemia of CKD in *Tmprss6*‐LKO and their littermate control mice with or without treatment of FG‐4592 (30 mg kg^−1^, once a day) for 4 weeks. B–J) RBC B), HGB C), HCT D), renal *Epo* mRNA E), serum Epo F), hepatic *Hamp* mRNA G), serum hepcidin H), serum iron I) and duodenal iron levels J) were detected in vehicle‐ and FG‐4592‐treated control and *Tmprss6*‐LKO mice with sham operation or 5/6Nx. K) Representative duodenum images of Hif2α, Hif1α, and Fpn immunohistochemistry from vehicle‐ and FG‐4592‐treated control and *Tmprss6*‐LKO mice with 5/6Nx. Scale bars, 100 µm. L) Duodenal *Fpn* mRNA levels were tested in vehicle‐ and FG‐4592‐treated control and *Tmprss6*‐LKO mice with sham operation or 5/6Nx. M) Representative western blot results of Hif2α, Fpn, and Dmt1 from vehicle‐ and FG‐4592‐treated control and *Tmprss6*‐LKO mice with 5/6Nx. Two‐way ANOVA with Tukey's post hoc test (for multi‐group comparisons), *n* = 4–11 mice per group. N) A schematic diagram illustrats the model that FG‐4592 promotes erythropoiesis in the high hepcidin context. FG‐4592 pharmacologically increases HIF in the duodenum by increasing the transcription levels of the HIF target gene *Fpn*, which increases iron delivery to marrow erythroblasts to increase erythropoiesis and thus improves anemia.

FG‐4592 has been reported to increase endogenous EPO and reduce hepcidin to improve anemia of chronic kidney diseases (CKD) in clinical trials.^[^ ^32^, ^33^ ^]^ To further test whether the duodenal HIF‐FPN pathway is also involved in FG‐4592′s effect on anemia of CKD, we randomly assigned *Tmprss6*‐LKO mice and littermate controls to sham‐operated and 5/6 nephrectomy (5/6Nx) groups to induce anemia of CKD, with or without FG‐4592 treatment (**Figure** **3**A). Higher serum creatinine and blood urea nitrogen (BUN) levels and stronger renal Masson's trichrome staining (Figure S5A–C, Supporting Information) and lower levels of RBC, HGB, and HCT were shown in untreated 5/6Nx mice compared to sham‐operated mice (Figure 3B–D), suggesting the anemia of CKD model was successfully established. Notably, FG‐4592 didn't affect renal injury‐related parameters in either 5/6Nx‐treated control or *Tmprss6*‐LKO mice (Figure S5A–C, Supporting Information). Consistent with previous findings,^[^ ^32^, ^33^ ^]^ FG‐4592 significantly ameliorates anemia of CKD. Notably, RBC, HGB and HCT levels in vehicle‐treated‐*Tmprss6*‐LKO‐5/6Nx mice were comparable to the sham‐operated*‐Tmprss6*‐LKO mice, but significantly improved in FG‐4592‐fed‐*Tmprss6*‐LKO‐5/6Nx mice (Figure 3B–D). Spleen mass, spleen histological assessments, renal *Epo*, serum Epo, *Erfe* in BM and spleen, hepatic *Hamp*, serum hepcidin and splenic non‐heme iron levels (Figure S5D–H, Supporting Information; Figure 3E–H) were not different between FG‐4592‐treated‐ and vehicle‐treated‐*Tmprss6*‐LKO‐5/6Nx mice, whereas increased serum iron levels (Figure 3I), unchanged hepatic iron levels (Figure S5I, Supporting Information) and decreased duodenal iron levels (Figure 3J) were observed in *Tmprss6*‐LKO‐5/6Nx mice upon FG‐4592 treatment. Coincidently, we observed higher duodenal Hif2α, Hif1α, unchanged *Dmt1* mRNA levels, significantly increased *Fpn* mRNA and protein levels (Figure 5K–M; Figure S5J,K, Supporting Information) in *Tmprss6*‐LKO‐5/6Nx mice upon FG‐4592 treatment. Thus, we conclude FG‐4592 accelerates the recovery from anemia of CKD, even at high hepcidin levels, by activating duodenal *Fpn* expression (Figure 3N).

### 2.4 FG‐4592 Mediates Iron Homeostasis in Acute and Chronic Inflammatory Conditions

**Figure 4:**
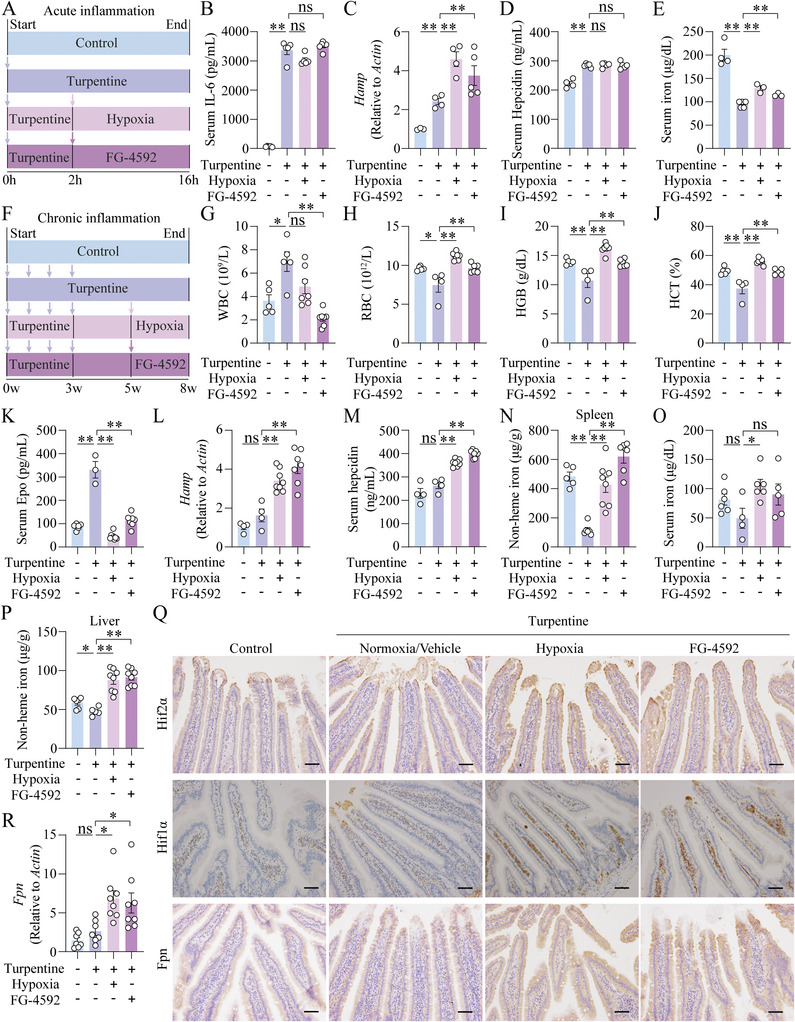
Hypoxia and FG‐4592 improve anemia phenotypes in acute and chronic TO‐induced anemia of inflammation in mice. A) A schematic diagram illustrats the experimental design of turpentine oil (TO, 5 ml kg^−1^)‐induced acute inflammation in 8‐week‐old wild‐type mice. After 2 h of TO induction, the mice were fed with FG‐4592 (30 mg kg^−1^) or placed in a hypoxia chamber for 14 h and all mice were sacrificed at 16 h after the first injection of vehicle or turpentine. B–E) Serum IL‐6 B), hepatic *Hamp* C), serum hepcidin D) and serum iron E) levels were detected in vehicle‐treated, TO‐treated, hypoxia‐exposed and TO‐treated, and FG‐4592‐fed and TO‐treated mice. F) A schematic diagram illustrats the experimental design of TO (5 ml kg^−1^, once a week, 3 weeks, 4 injections in total)‐induced chronic AI in 8‐week‐old wild‐type mice. After 2 weeks post the last injection of TO, the mice were placed in a hypoxia chamber or gavaged with FG‐4592 (30 mg kg^−1^, once a day) for 3 weeks. G–P) WBC G), RBC H), HGB I) and HCT J), serum Epo K), hepatic *Hamp* mRNA L), serum hepcidin M), splenic iron N), serum iron O) and hepatic iron levels P) were detected in vehicle‐treated, TO‐treated, hypoxia‐exposed and TO‐treated, and FG‐4592‐fed and TO‐treated mice. Q,R) Representative duodenum images of Hif2α, Hif1α, and Fpn immunohistochemistry Q), and duodenal *Fpn* mRNA levels R) were shown in the four groups of mice in the chronic AI model. *n* = 4–8 mice per group. Scale bars, 100 µm. One‐way ANOVA with Tukey's post hoc test (for multi‐group comparisons).

AI is defined as normocytic anemia with systemic inflammation and elevated hepcidin levels,^[^ ^3^ ^]^ which is prevalent in patients with infections, malignancies, or autoimmune disorders. To investigate the effect of hypoxia or FG‐4592 on AI, mice were injected with a single dose of turpentine oil (TO) to establish a widely used mouse model of acute‐phase inflammation (**Figure** **4**A).^[^ ^34^, ^35^ ^]^ Early after a single dose of TO injection (at the 16^th^ hour), serum IL‐6 levels, hepatic *Hamp* and serum hepcidin levels were significantly higher (Figure 4B–D) compared to the vehicle‐injected controls. Interestingly, hepatic *Hamp* and serum hepcidin levels failed to decrease after hypoxia or FG‐4592 treatment for 14 h post TO‐treatment compared to the untreated TO group (Figure 4C,D). Serum iron in the TO‐treated group was lower than in vehicle‐injected controls, whereas increased significantly upon either hypoxia or FG‐4592 treatment (Figure 4E), consistent with upregulated duodenum Fpn levels (Figure S6, Supporting Information).

In addition, mice were injected weekly with TO over 3 weeks to induce AI, followed by a 2‐week recovery period and treated with either hypoxia or FG‐4592 for an additional 3 weeks (Figure 4F). The increased WBC counts reflected the existence of chronic inflammation (Figure 4G). Greater anemia‐related blood parameters were presented after hypoxia or FG‐4592 treatment compared to untreated TO‐induced chronic anemia (Figure 4H–J). Mice chronically exposed to TO showed higher levels of renal *Epo* and serum Epo, which were decreased by hypoxia or FG‐4592 treatment (Figure S7A, Supporting Information; Figure 4K). *Erfe* levels in BM and spleen were unchanged (Figure S7B,C, Supporting Information). Additionally, hepatic *Hamp* and serum hepcidin levels were significantly increased (Figure 4L,M), accompanied with increased levels of splenic iron, serum iron, and hepatic iron (Figure 4N–P), upon hypoxia or FG‐4592 treatment, compared to TO‐treated group. Importantly, stronger IHC staining of duodenal Hif2α, Hif1α, *Fpn* mRNA and protein levels were observed in hypoxia‐exposed or FG‐4592‐treated mice (Figure 4Q,R). And only hypoxia‐treated mice showed higher *Dmt1* levels (Figure S7D, Supporting Information). These results suggest hypoxia and FG‐4592 treatment can improve chronic inflammation‐induced anemia mainly by increasing duodenum *Fpn* expression.

### 2.5 FG‐4592 Accelerates Recovery from 5‐Fluorouracil‐Induced Anemia

**Figure 5:**
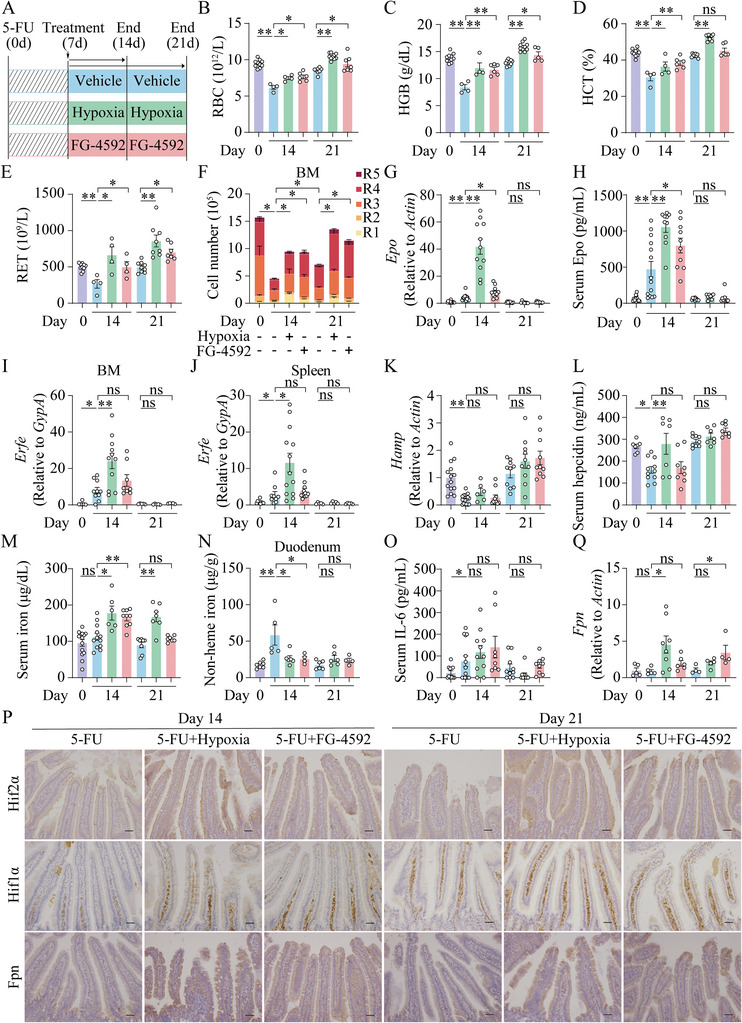
FG‐4592 or hypoxia treatment improves 5‐fluorouracil‐induced chemotherapy‐related anemia. A) A schematic diagrasm illustratsings the experiment design of 8‐week‐old wild‐type mice treated with 5‐FU (150 mg kg^−1^) to model CRA. As indicated, the treated mice were sacrificed on either day 14 or day 21 after the first injection of 5‐FU. Mice were randomly placed in either a hypoxia chamber or gavaged with FG‐4592 (30 mg kg^−1^, once a day) for one week from day 7 to day 14 after the first injection of 5‐FU; or randomly assigned to either a hypoxia chamber or gavaged with FG‐4592 (30 mg kg^−1^, once a day) for two weeks from day 7 to day 21 after the first injection of 5‐FU. B–E) RBC B), HGB C), HCT D) and RET E) in vehicle‐treated, 5‐FU‐treated, hypoxia‐exposed and 5‐FU‐treated, and FG‐4592‐fed and 5‐FU‐treated mice. F) Flow cytometry analysis of erythroid cell populations (R1–R5) in bone marrow at day 0, 14, and 21 post 5‐FU injection. R2‐R5: day 14 of 5‐FU group versus control group; R2, R4‐R5: day 14 of 5‐FU group versus day 21 of 5‐FU group; R1‐R5: comparison between hypoxia/FG‐4592 treatment at day 14 and 21 group, and untreated 5‐FU group at day 14 and 21, respectively. G–O) Renal *Epo* mRNA G), serum Epo H), *Erfe* mRNA normalized to *GypA* in the bone marrow I) and spleen J), hepatic *Hamp* mRNA K), serum hepcidin L), serum iron M), duodenal iron N) and serum IL‐6 O) levels were measured in the indicated groups. P) Representative images of immunohistochemistry for duodenal Hif2α, Hif1α, and Fpn from the indicated groups at day 14 and day 21. Scale bars, 100 µm. Q) Duodenal *Fpn* mRNA levels were tested in the indicated groups. *n* = 4–15 mice per group.One‐way ANOVA with Tukey's post hoc test (for multi‐group comparisons).

Anemia remains a common complication of cancer therapy.^[^ ^36^ ^]^ To evaluate whether hypoxia or FG‐4592 modulates the responses to chemotherapy‐related anemia (CRA), mice were subjected to the antimetabolite 5‐fluorouracil (5‐FU), a widely used chemotherapeutic drug to induce a severe and persistent anemia.^[^ ^37^ ^]^ We compared anemia recovery time with or without hypoxia exposure or FG‐4592 treatment on days 14 and 21 after the initiation of 5‐FU treatment (**Figure** **5**A). The recovery from anemia was accelerated in hypoxia‐ or FG‐4592‐treated mice compared to untreated 5‐FU‐injected mice, respectively (Figure 5B–E). In addition, we found that in the BM, the cell counts of R1 were unchanged, but R2‐R5 decreased significantly at day 14, while R2, R4, and R5 recovered at day 21 upon 5‐FU injection (Figure 5F; Figure S8A,B, Supporting Information). Notably, either hypoxia or FG‐4592 treatment for 7 days could significantly increase R1 numbers and continue the recovery of R2‐R5 cell populations upon 5‐FU injection at days 14 and 21 (Figures 5F; S8A,B, Supporting Information).

Interestingly, hepatic *Hamp* and serum hepcidin levels were increased significantly at day 7, suggesting 5‐FU‐induced CRA belongs to hepcidin‐activated anemia (Figure S8C,D, Supporting Information). In addition, hepatic *Hamp* and serum hepcidin levels were decreased at day 14 and back to normal at day 21 post 5‐FU injection, accompanied by higher levels of renal *Epo*, serum Epo, *Erfe* in BM and spleen at day 14 upon 5‐FU injection (Figure S8C,D, Supporting Information; Figure 5G–L). Importantly, despite higher levels of renal *Epo* and serum Epo at day 14 upon FG‐4592 or hypoxia treatment and higher *Erfe* at day 14 upon hypoxia treatment, compared to the untreated 5‐FU‐injected group, hepatic *Hamp* and serum hepcidin levels were unchanged or even higher upon hypoxia or FG‐4592 treatment at either day 14 or day 21 (Figure 5G–L). However, the levels of serum iron and hepatic iron were increased at day 14 upon hypoxia or FG‐4592 treatment and at day 21 upon hypoxia treatment (Figures 5M; S8E, Supporting Information), compared to the simple 5‐FU injected group at day 14 or 21, respectively. Besides, duodenal iron levels increased at day 14 post 5‐FU treatment but recovered at day 21 post 5‐FU treatment (Figure 5N).

Of note, hypoxia or FG‐4592 treatment for 7 days could significantly decrease duodenal iron retention (Figure 5N). Besides, elevated serum IL‐6 indicates 5‐FU‐induced anemia is accompanied by inflammation (Figure 5O). Most importantly, hypoxia or FG‐4592 intervention rescued 5‐FU‐induced anemia without altering inflammation (Figure 5O). Consistently, hypoxia or FG‐4592 treatment exhibited greater protein expression of Hif2α, Hif1α (Figure 5P). Both 7 days of hypoxia and 14 days of FG‐4592 treatment could significantly upregulate duodenal *Fpn* mRNA levels compared with untreated‐5‐FU controls, respectively (Figure 5Q). However, only hypoxia treatment for 7 days increased duodenal *Dmt1* mRNA levels (Figure S8F, Supporting Information). And duodenal Fpn protein levels were increased upon hypoxia or FG‐4592 treatment, compared to untreated 5‐FU‐injected mice (Figure 5P). Taken together, these results suggest hypoxia and FG‐4592 treatment can significantly improve 5‐FU‐induced anemia by increasing duodenum *Fpn* expression in a hepcidin‐independent manner.

### 2.6 FG‐4592 Improves IRIDA Mainly through HIF2α Upregulated Duodenal *FPN* Expression

**Figure 6:**
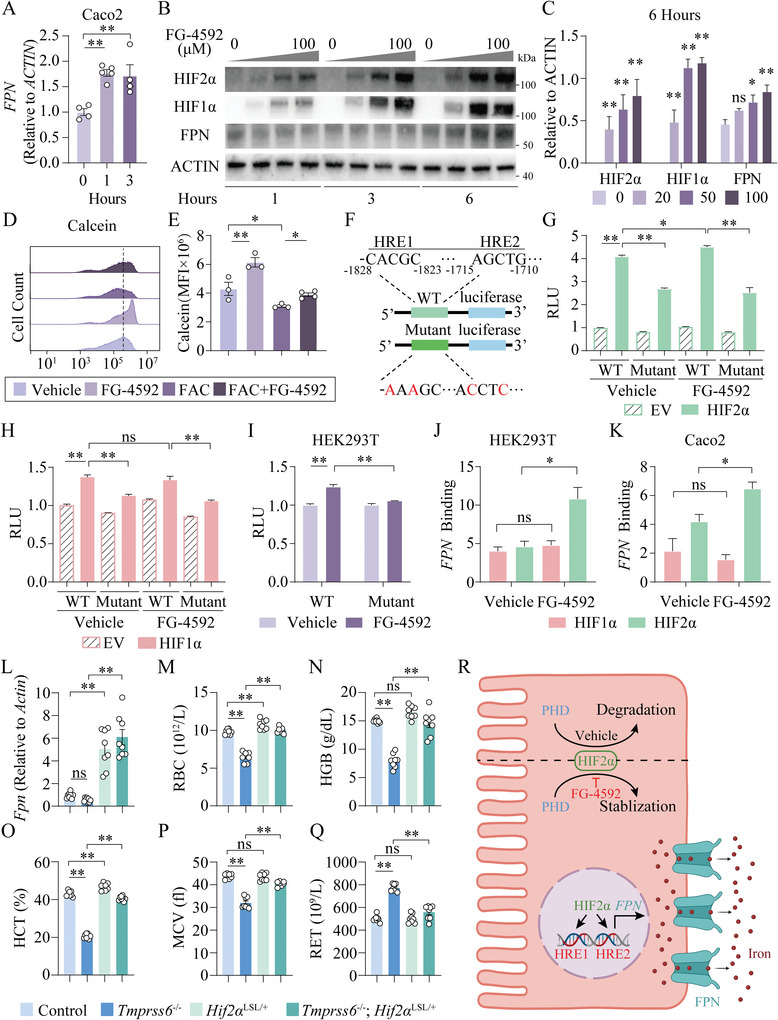
FG‐4592 upregulates *FPN* expression via HIF2α binding to HREs. A) Real‐time qPCR analysis of *FPN* levels in Caco2 cells treated with FG‐4592 at 0, 1, and 3 h. B,C) Representative western blot analysis of HIF2α, HIF1α, and FPN levels in Caco2 cells treated with FG‐4592 (0, 20, 50, 100 µM) for 1, 3, and 6 h B), and quantitation for the results of 6‐h time points, results were shown relative to ACTIN and comparison between 20/50/100 µm with 0 µm C). D,E) Flow cytometry analysis D) and summary of Calcein‐AM mean fluorescence intensity (MFI) E) in Caco2 cells treated with FG‐4592 for 24 h, with or without ammonium ferric citrate (FAC:100 µm) treatment. F) A schematic diagram of the mouse *Fpn* promoter illustrating two wild‐type or mutant HREs in the regulatory region, which are numbered in relation to the translation initiation site. G,H) HEK293T cells were transiently transfected with wild‐type mouse *Fpn* or the mutant luciferase‐reporter construct, and co‐transfected with empty vector or Hif2α G) or Hif1α H) expression plasmids or treated with FG‐4592. I) Relative light units (RLU) of reporter activity in HEK293T cells that were transiently transfected with the wild‐type or the mutant luciferase construct, and treated with FG‐4592. J,K) Chromatin immunoprecipitation (ChIP) assay results from HEK293T and Caco2 cells expressing HIF1α and HIF2α relative to normal anti‐rabbit IgG treated with FG‐4592. L–Q) In control, *Tmprss6* ^−/−^, *Hif2α* ^LSL/+^ and *Tmprss6* ^−/−^ *; Hif2α* ^LSL/+^ mice, duodenal *Fpn* mRNA levels L), RBC M), HGB N), HCT O), MCV P), and RET Q) were measured, respectively. (*n* = 7–8 mice per group). A, E, G‐Q, one‐way ANOVA with Tukey's post hoc test. R) A schematic diagram illustrats that FG‐4592 functions as a stabilizer of duodenal HIF2α, which binds to two HREs of the *FPN* promoter to stimulate *FPN* expression and thus promotes duodenal iron export.

To further explore the mechanisms underlying FG‐4592‐mediated regulation of Fpn expression, we treated Caco‐2 cells, a widely used human intestinal epithelial cell line, with FG‐4592. *FPN* mRNA levels were significantly greater at the 1 h‐ and 3 h‐time points post FG‐4592 treatment compared to the 0 h‐time point (**Figure** **6**A), suggesting FG‐4592 regulates *FPN* expression at the transcriptional level. Besides, the protein levels of HIF2α, HIF1α, and FPN were significantly greater in a dose‐dependent manner at the 6 h after FG‐4592 treatment compared to untreated control cells (Figure 6B,C). To measure intracellular iron levels, we used the iron‐sensitive fluorophore Calcein‐AM, which is quenched upon binding iron.^[^ ^38^ ^]^ Intracellular iron levels were significantly lower at 24 h post FG‐4592 treatment compared to respective controls, either with or without ferric citrate (FAC) treatment (Figure 6D,E), consistent with upregulated FPN levels.

In the promoter of the *Fpn* gene, there are two putative hypoxia response elements (HREs).^[^ ^29^ ^]^ To further test whether FG‐4592 regulates *Fpn* transcription through directly modulating these two HREs, we analyzed the wild‐type Fpn promoter and its HRE site‐specific mutants by the dual‐luciferase reporter assay (Figure 6F). Co‐transfection with a mammalian Hif2α expression construct could strongly increase the luciferase activity of *Fpn* in HEK293T cells compared to those of empty vector controls (Figure 6G). Whereas the putative HRE mutants showed a significant inhibitory‐effect on *Fpn* expression upon Hif2α induction, which functionally validated these HREs sites were specific HIF‐binding sites (Figure 6G). Interestingly, FG‐4592 upregulated *Fpn* expression upon Hif2α‐mediated induction (Figure 6G). To a lesser extent, similar effects were also observed when co‐transfected with a Hif1α expression construct (Figure 6H). However, FG‐4592 didn't further change *Fpn* expression upon Hif1α‐mediated induction (Figure 6H). Importantly, FG‐4592 could directly upregulate the luciferase activity of *Fpn* through HREs (Figure 6I). Consistently, the chromatin immunoprecipitation (ChIP) assay showed increased binding activity of HIF2α at the *FPN* promoter at 3 h post FG‐4592 treatment, but not of HIF1α in both HEK293T and Caco2 cells (Figure 6J–K).

To further examine whether Hif2α activation can effectively alleviate hepcidin‐activated anemia, we crossed *Tmprss6^−/−^* mice with intestine‐specific *Hif2α* overexpression mice (*Hif2α* ^LSL/+^) to generate *Tmprss6* ^−/−^; *Hif2α* ^LSL/+^ mice. Interestingly, *Tmprss6* ^−/−^; *Hif2α* ^LSL/+^ mice showed significantly higher duodenal *Fpn* mRNA levels, and importantly, fully rescued blood routine indexes compared to *Tmprss6* ^−/−^ mice (Figure 6L–Q). In addition, we found that PT2385 (the Hif2α specific inhibitor)^[^ ^39^ ^]^ treatment could counterbalance the protective effects of hypoxia on *Tmprss6^−/−^* mice (Figure S9, Supporting Information). Taken together, these data demonstrate FG‐4592 treatment increases *FPN* expression mainly through HIF2α binding to the HREs on the promoter of *FPN* (Figure 6R), and overexpressing duodenal Hif2α could directly trigger *Fpn* expression and rescue hepcidin‐activated anemias.

### 2.7 Intestinal *Hif2α* Is Required for the Recovery from 5‐FU‐Induced Anemia in Mice

**Figure 7:**
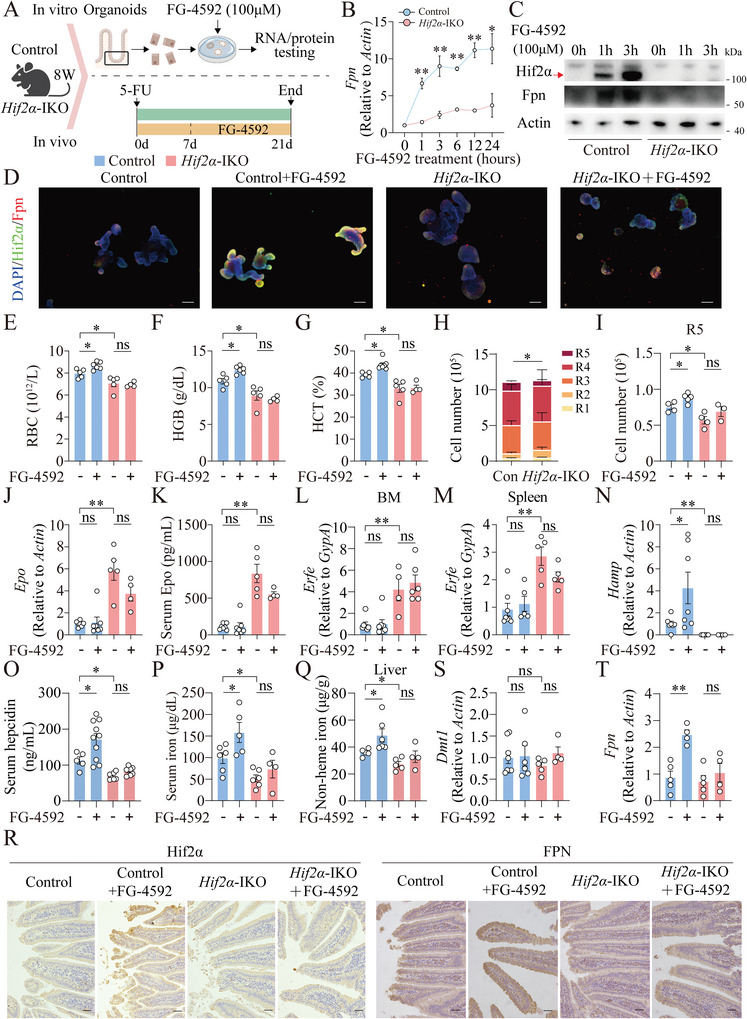
Intestine‐specific *Hif2α* knockout reduces recovery from 5‐fluorouracil‐induced chemotherapy‐related anemia. A) A schematic diagram illustrats the use of control and *Hif2α*‐IKO to establish in vitro intestinal organoids, that were treated with 100 µM FG‐4592 within 24 h for RNA measurement and 1–3 h for protein testing. Additionally, control and *Hif2α*‐IKO mice were injected with 5‐FU, and fed with vehicle or FG‐4592 for 14 days. B–D) *Fpn* mRNA B), Hif2α and Fpn protein C), and immunofluorescent staining for Hif2α and Fpn D) from the organoids described in A） were detected. E–G) RBC E), HGB F), and HCT G) levels in littermate controls and *Hif2α*‐IKO mice at day 21 after 5‐FU injection, with or without daily FG‐4592 treatment 7 days after 5‐FU injection. H,I) Flow cytometry analysis of R1‐R5 in bone marrow of control and *Hif2α*‐IKO mice at day 21 of 5‐FU injection with or without FG‐4592 treatment from day 7 to day 21 after 5‐FU injection. H): * represents *p* < 0.05 in R5 between 5‐FU treated control and *Hif2α*‐IKO mice. J–Q) Renal *Epo* mRNA J), serum Epo K), *Erfe* mRNA normalized to *GypA* in the bone marrow L) and spleen M), hepatic *Hamp* mRNA N), serum hepcidin O), serum iron P) and liver iron levels Q) were measured in 5‐FU administrated control and *Hif2α*‐IKO mice with or without FG‐4592 treatment. R) Representative images of immunohistochemistry for Hif2α and Fpn in duodenal sections from the indicated four groups. Scale bars, 100 µm. S,T) Duodenal *Dmt1* S) and *Fpn* mRNA T) were detected in the indicated four groups. *n* = 4–10 mice per group. Two‐way ANOVA with Tukey's post hoc test (for multi‐group comparisons).

To confirm the effect of FG‐4592 on Hif2α‐mediated *Fpn* expression, we generated intestinal‐specific *Hif2α* knockout mice (*Hif2α‐*IKO) by crossing *Hif2α^flox/flox^* mice with *Villin‐Cre* mice (Figure S10A, Supporting Information), and examined the function of duodenal *Hif2α* by cultured intestinal organoids in vitro and 5‐FU administration in vivo as indicated (**Figure** **7**A). Unlike control organoids upon FG‐4592 treatment, upregulation of *Fpn* expression (Figure 7B), and Hif2α as well as Fpn protein levels (Figure 7C), were significantly blunted in *Hif2α‐*IKO‐derived intestinal organoids. And Hif2α has the expression pattern similar to Fpn, both could be enhanced mainly in the basolateral of control organoids treated with FG‐4592 (Figure 7D; Figure S10B, Supporting Information). Besides, *Hif2α‐*IKO mice showed significantly delayed recovery from 5‐FU‐induced anemia at day 21 compared to littermate controls (Figure 7E–G). Consistently, *Hif2α‐*IKO mice had significantly lower levels of R5 at day 21 in the BM (Figure 7H), and *Hif2α* is required for the effectiveness of FG‐4592 in improving R5 levels in 5‐FU‐induced anemia (Figure 7I). Moreover, other than similar inflammation levels (Figure S10C, Supporting Information), 5‐FU‐treated *Hif2α‐*IKO mice had significantly higher levels of renal *Epo*, serum Epo, *Erfe* in BM and spleen, and lower levels of hepatic *Hamp*, serum hepcidin, serum iron, and hepatic iron, compared to those of control mice (Figure 7J–Q). Importantly, the rescue effects of FG‐4592 on anemia‐related and iron‐related parameters, especially duodenal Hif2α, *Fpn* mRNA, and protein levels in 5‐FU treated control mice could not be replicated in *Hif2α‐*IKO mice (Figure 7R–T; Figure S10D, Supporting Information). In comparison, loss of intestinal *Hif1α* didn't affect the degree of 5‐FU‐induced anemia (Figure S11, Supporting Information). Taken together, these data demonstrate an indispensable role for an intestinal Hif2α‐Fpn axis in the protective activity of FG‐4592 against perturbed iron absorption‐related anemias.

## 3 Discussion

In this study, we report hypoxia treatment or its pharmacologically mimetic drug that improves hepcidin‐activated anemias, including IRIDA, CKD‐associated anemia, AI, and CRA. It is well‐known that HIFs are the master regulators of cellular adaptation to hypoxia. A previous study reported that Hif1α could directly repress *Hamp* transcription.^[^ ^40^ ^]^ Additionally, HIFs can suppress *HAMP* via inducing renal EPO synthesis and subsequently promoting ERFE synthesis in the spleen and BM.^[^ ^41^, ^42^, ^43^ ^]^ However, this regulatory mechanism doesn't apply to these hepcidin‐activated anemia scenarios. Instead, we functionally characterize here that targeting the duodenal Hif2α‐Fpn axis could directly promote iron absorption independent of hepcidin, especially in hepcidin‐activated and iron‐refractory anemias.

To investigate its potential translational applications, we examined the efficacy of FG‐4592 in animal models of hepcidin‐activated anemias. FG‐4592 functions by stabilizing HIFs through the potent inhibition of PHD activity. Notably, among the available PHD inhibitors, FG‐4592 has received clinical approval for the treatment of anemia of CKD in certain countries, such as China and Japan. Previous studies showed FG‐4592 improved anemia of CKD by increasing Epo and reducing serum hepcidin in the patients.^[^ ^32^, ^33^ ^]^ Interestingly, we found intestine serves as the additional important effective organ of FG‐4592, which functions to activate duodenal *Fpn* expression by stabilizing Hif2α. This defines an additional important compensatory mechanism in the intestine that underlies the hypoxia/FG‐4592‐induced accelerated recovery from hepcidin‐activated anemias in the murine models, consistent with the fact that mice derive a greater proportion of their daily iron needs from dietary intake versus erythroid turnover.^[^ ^44^ ^]^ In addition, Hif2α is sensitive to cellular iron and oxygen levels, and regulated by the hepcidin‐Fpn axis through limiting the activity of iron‐dependent PHD enzymes.^[^ ^23^ ^]^ In cases of hepcidin‐activated anemias, hepcidin levels were increased accompanied by decreased duodenal Fpn expression and accumulated duodenal iron. This dysregulation could ultimately result in suppressed Hif2α expression. Consequently, there is a lack of an appropriate intestinal HIF response, which is a major mechanism underlying the development of anemia. Therefore, targeting Hif2α in the duodenum through hypoxia or FG‐4592 treatment may offer a potential mechanism to bypass iron regulation in these conditions.

Although Dmt1 has been previously shown to be a Hif2α target gene under low iron conditions,^[^ ^21^ ^]^ using other stimuli such as PHD inhibition or hypoxia, the duration, concentration, and extent of the treatment could lead to variable results with Dmt1 in vivo, unlike what is seen during iron deficiency. Notably, the transcriptional regulation of *Fpn* by Hif2α has also been well described,^[^ ^20^, ^22^, ^29^ ^]^ these important findings have created new therapeutic opportunities for the treatment of hemochromatosis and anemia. For example, intestinal Hif2α‐Fpn axis is essential for the local absorptive response to systemic iron overload. Deletion of intestinal Hif2α or pharmacological blockade of Hif2α using a clinically relevant inhibitor PT2385 successfully reduced iron accumulation in a mouse model of hepcidin‐deficient hemochromatosis.^[^ ^23^, ^45^ ^]^ Besides, intestine‐specific disruption of *Hif2α* ^[^ ^46^, ^47^ ^]^ or dietary iron restriction^[^ ^48^ ^]^ or use of the oral Fpn inhibitor vamifeport^[^ ^49^, ^50^, ^51^ ^]^ has been reported to improve iron‐overloaded anemias, such as sickle cell disease and β‐thalassemia in mice. In this study, we functionally demonstrate stabilizing duodenal Hif2α by hypoxia or FG‐4592 treatment results in significantly increased *Fpn* expression and subsequent improvement of many types of anemias. Therefore, targeting the duodenal Hif2α‐Fpn axis serves as a promising therapeutic strategy for modulating perturbed iron absorption‐related pathogenic conditions. One of the limitations of our study is that inducible intestinal *Fpn* or *Dmt1* knockout mice might be further tested for exploring the precise mechanism of FG‐4592.

From a clinical perspective, hepcidin‐activated anemia remains a highly prevalent morbidity in patients with chronic inflammation, posing a major challenge to the development of treatment strategies. In principle, RBC transfusions, iron supplementation and using erythropoiesis‐stimulating agents (ESAs)^[^ ^3^ ^]^ are three treatment options for AI. However, RBC transfusions are restricted to patients with severe anemia (Hb < 8 g L^−1^) and are considered as a temporary strategy due to increased mortality in specific conditions.^[^ ^52^, ^53^ ^]^ Accordingly, iron supplementation is also ineffective in this case, as iron treatment could further increase not only hepcidin levels but also the pathogenicity of some microbes, which in turn increased the burden of infectious diseases,^[^ ^3^ ^]^ ESAs, like epoetin or darbepoetin alfa, are associated with cardiovascular side effects and increased risks for thrombosis.^[^ ^54^ ^]^ Notably, loss of *Tmprss6* presents with a drastically blunted hepcidin responsiveness upon Epo treatment.^[^ ^55^ ^]^ Therefore, there is an urgent need to develop novel safe and effective therapies for hepcidin‐activated anemias. Our findings functionally demonstrate the efficacy of FG‐4592 as a novel approach for the treatment of IRIDA, AI, and CRA. However, one limitation is that FG‐4592 treatment could trigger both iron absorption pathways and other metabolism pathways,^[^ ^56^ ^]^ we could not completely rule out other potential off‐target effects of FG‐4592.

## 4 Conclusion

In conclusion, the hepcidin independent effect of Hif2α‐Fpn axis on anemia recovery was demonstrated in multitype of anemia models, including IRIDA, inflammatory anemia, and chemotherapy‐induced anemia. Importantly, this study provides compelling evidence of a clinically relevant pharmacological approach to target the duodenal Hif2α‐Fpn axis as a novel strategy to improve various forms of refractory hepcidin‐activated anemias.

## 5 Experimental Section

### Animal Models

*Tmprss6^flox/+^* mice (Shanghai Biomodel Organism, China) were constructed by deleting exon 3 of the *Tmprss6* gene, then backcrossed to the C57BL/6J background for over ten generations (Figure S1A, Supporting Information). The pure background *Tmprss6^flox/+^* mice were crossed with either *CMV*‐Cre or *Alb*‐Cre transgenic mice to generate either global or hepatocyte‐specific *Tmprss6* knockout mice, respectively. Either *Hif2α^flox/flox^* mice (GemPharmatech Co. Ltd., China), *Hif1α^flox/flox^* mice (Southern Medical University), or *Hif2α^LSL/+^* mice^[^ ^57^, ^58^ ^]^ were crossed with *Villin*‐Cre transgenic mice to generate intestine‐specific *Hif2α*‐ or *Hif1α*‐knockout or *Hif2α* overexpression mice, respectively. All mice were fed a standard AIN‐76A diet containing 50 mg iron kg^−1^ (Research Diets, Inc). All animal experiments were approved by the Institutional Animal Care and Use Committee of Zhejiang University.

### Statistical Analysis

Data were presented as the mean ± standard error of mean. The sample size for each statistical analysis was provided in the figure legends. Differences between groups were analyzed using a two‐tailed, unpaired Student *t*‐test (for two groups), or one‐ or two‐way ANOVA with Tukey's post hoc test (for multi‐group comparisons), where indicated. * represents *p* <0.05; ** represents *p* <0.01; ns represents not significant. All statistical analyses were performed using GraphPad Prism version 8.

### Procedures and Methods

Details regarding protocols and methods are provided in the Supporting Information.

### Data Sharing Statement

Data are available on reasonable request. Please contact Professor Fudi Wang (fwang@zju.edu.cn).

## Conflict of Interest

The authors declare no conflict of interest.

## Author Contributions

Y.Y., Y.S., and S.Y. contributed equally to this work. F.W., J.M., Y.Y., Y.S., S.Y., and Y.M.S designed the experiments. All authors acquired and analyzed the data. Y.Y., Y.S., and S.Y. performed the statistical analyses. Y.L., Z.L., N.K.D., Q.W., J.Z., S.S., X.L., and W.Y. assisted murine experiments. Y.Y., J.M., and F.W. drafted the manuscript, and Y.Y., J.M., F.W., Y.S., S.Y., and Y.M.S. revised the manuscript. F.W. and J.M. obtained funding and supervised the study. All authors approved the final version of the paper.

## Supporting information

Supporting Information

## Data Availability

The data that support the findings of this study are available from the corresponding author upon reasonable request.
