# Supplementary material for: Activation of Intestinal HIF2α Ameliorates Iron‐Refractory Anemia
Source: Adv Sci (Weinh). 2024 Jan 20;11(12):2307022. doi: 10.1002/advs.202307022 (PMC10966566; doi:10.1002/advs.202307022)
Supplement: Supplementary file 1 — Supporting Information [file ADVS-11-2307022-s001.pdf]

ADVANCED SCIENCE
Open Access

Supporting Information

for *Adv. Sci.*, DOI 10.1002/advs.202307022

Activation of Intestinal HIF2α Ameliorates Iron-Refractory Anemia

Yingying Yu, Yunxing Su, Sisi Yang, Yutong Liu, Zhiting Lin, Nupur K. Das, Qian Wu, Jiahui Zhou, Shumin Sun, Xiaopeng Li, Wuyang Yue, Yatrik M. Shah, Junxia Min* and Fudi Wang**

Online Supplemental Data

Activation of Intestinal HIF2α Ameliorates Iron-Refractory Anemia

Yingying Yu, Yunxing Su, Sisi Yang, Yutong Liu, Zhiting Lin, Nupur K. Das, Qian Wu, Jiahui Zhou, Shumin Sun, Xiaopeng Li, Wuyang Yue, Yatrik M. Shah, Junxia Min& Fudi Wang

This file includes:

Supplemental Methods

Supplemental Table 1-4

Supplemental Figure 1-11

Supplemental Methods

Genotyping of *Tmprss6*$^{-/-}$, *Tmprss6*-LKO, *Hif1α*-IKO, and *Hif2α*-IKO mice

Using EasyDo Genomic DNA Kit (EasyDo, Hangzhou, China) to extract genomic DNA from mouse tail biopsies, then using 2×HiFiTaq PCR StarMix with Loading Dye, polymerase chain reaction (PCR) primers and extracted genomic DNA to prepare the PCR mixture. The samples were then subjected to electrophoresis using 1.2% agarose gel spiked with green blue nucleic acid dye at a constant pressure of 120V for 30 mins and finally imaged by a chemiluminescent gel imaging system. The primers are listed in Supplemental Table 1.

Flow cytometry analysis

Cells from bone marrow were isolated using cold PBS and filtered through a 70 μm cell strainer, then 4°C centrifuged at 300 x *g* for 5 minutes to remove the supernatant. For detection of erythroid cells, cells were stained with the mixed flow cytometric antibody: DAPI (422801, Biolegend, 1:1000), APC/Cyanine7 anti-mouse CD45 Antibody (103115, Biolegend, 1:200), APC anti-mouse TER-119/Erythroid Cells Antibody (116211, Biolegend, 1:200), PE anti-mouse CD44 Antibody (103024, Biolegend, 1:200), and incubated at 4°C shaker for 30 minutes, then washed with cold PBS, centrifuged and finally resuspended with 300 μL of cold PBS. For detecting intracellular iron levels, the Caco2 cells were incubated with or without the addition of 2,2′-bipyridine (a high-affinity iron chelator), washed, and then incubated with clacein-AM (final concentration: 5 μM) for 10 mins at 37°C. All samples were analysed using an ACEA NovoCyteTM (ACEA Biosciences, United States) flow cytometric analyser.

Real-time quantity polymerase chain reaction (RT-PCR) analysis

Total RNA was extracted from the tissue or cells using TransZol Up (TransGen Biotech, Beijing, China) and reverse transcripted using the ABScript III RT Master Mix for qPCR (ABclonal. Wuhan, China) to obtain cDNA, then real-time fluorescent PCR samples were prepared using Hieff UNICON Universal Blue qPCR SYBR Green

Master Mix (Yeasen Biotech, Shanghai, China). All the procedure was followed by the manufacturer's instruction. The resulting samples were assayed using the Roche LightCycler® 480 II (Roche, Shanghai, China) and analysed using the Abs 2nd. The primers used are listed in the supplemental Table 2.

Western blot analysis

Total protein was extracted from cells using lysate which contains RIPA, PI and PMSF. The mixture was centrifuged at 12000 rpm for 10 minutes under 4°C. Supernatant was taken from the sample and added 5 x SDS-PAGE (Genestar, Beijing, China), then incubated in a metal bath at 37°C for 30 mins, centrifuged at 12000 rpm for 5 minutes under 4°C. The samples were then under electrophoresis (80V constant voltage, 90 minutes) and transfer (300mA constant current, 90 minutes) using electrophoresis and transfer system (Bio-rad). After blocked with 5% skimmed milk powder dissolved in TBST for 2 hours, the membrane was incubated using the primary antibody at 4°C on a shaker overnight, then washed. Incubated for 2 hours on a shaker using the secondary antibody, washed on the second day. Detected on ChemiDoc XRS+ Chemiluminescent imaging systems (Bio-Rad, United States) using FDbio-Pico FD8000 enhanced chemiluminescence (FDbio science, Hangzhou, China). Images were captured, optimized and analyzed by Quantity One Software. The primary antibodies used in Western Blot analysis are described below: anti-Ferroportin (Novus Biologicals, NBP1-21502, 1:1000) for human lysates, anti-Ferroportin (MTP11-A, Alpha Diagnostics, 1:1000) for mouse tissue lysates, anti-HIF1α (Proteintech, 66730-1-Ig, 1:1000) for human & mouse lysates, anti-HIF2α (Abcam, ab109616, 1:1000) for human & mouse lysates, anti-β-actin (Abclonal, A17910, 1:10000) for human & mouse lysates, anti-DMT1 (NRAMP21-A, ADI, 1:1000) for mouse tissue lysates.

Histological analysis and Immunohistochemistry

After fixed in 4% formaldehyde, kidneys and small intestines were fixed in paraffin. Macroscopic assessments of kidneys were performed on Masson's trichrome-stained sections. Small intestines were performed with the primary antibodies used in

immunohistochemistry analysis, including anti-Ferroportin (Alpha Diagnostics, 1:300), anti-HIF1α (Proteintech, 66730-1-Ig, 1:300), anti-HIF2α (Abcam, ab109616, 1:300).

Measurements of Tissue non-heme iron

The measurements were finished as previously described[1]. Mouse organ biopsies were weighed, then digested for 72 hours using a prepared acid solution. During this period, vortexed the sample every day. The samples were centrifuged after full digestion and then the reaction was carried out by adding the prepared colour solution and the absorbance was measured. The data was presented as micrograms of iron per gram wet weight of tissue.

Measurements of Serum iron and TIBC parameters

Centrifuge blood at 1.0x *g* for 10min, then take the supernatant for analyse. Serum iron concentration and UIBC were measured using a serum iron/TIBC kit (Pointe Scientific, Inc.) in accordance with the manufacturer's instructions. Transferrin saturation was calculated as (serum iron/TIBC x 100) with TIBC calculated from UIBC.

Measurements of serum Hepcidin, erythropoietin (Epo) and interleukin-6 (IL-6)

Mouse serum Hepcidin was assayed by enzyme-linked immunosorbent assay (ELISA) (Abcam; Cambridge, MA, Intrinsic Life Sciences, LaJolla, CA; SKU#HMC-001). Mouse serum Epo and IL-6 were detected using mouse Epo ELISA Kit and interleukin-6 ELISA kit (Elabscience, Wuhan, China) according to the manufacturer's instructions.

Measurements of serum Creatinine and Bun

Mouse serum creatinine and Bun were detected using assay kits (C011-2-1 and C013-2-1, respectively, from Nanjing Jiancheng, China) according to the manufacturer's instructions.

Hematological parameters

Peripheral blood was obtained and analyed using the hematology analyzer (Siemens)

at the Center for Drug Safety Evaluation and Research, Zhejiang University.

Chromatin immunoprecipitation (ChIP) assay

Caco2 and HEK293T cells were treated with vehicle or 100 μM FG-4592 for 3 hours and crosslinked in 1% formaldehyde in 1X PBS for 20 min. Nuclei were isolated and lysed in an SDS lysis buffer (50mM Tris-HCl pH 8.1, 10 mM, EDTA, 1% SDS, and protease inhibitors) according to the manufacturer's instructions (Cell Signaling Technology, #9005). Sheared soluble chromatin was immunoprecipitated with primary antibody for HIF1α or HIF-2α. Decrosslinked samples were incubated with RNase A and proteinase K and 1 μL of purified DNA was used for qPCR with primers listed in Supplementary Table 3. Antibody for ChIP: anti-HIF1α (Abcam, ab228649, 1:100), anti-HIF2α (Abcam, ab243861, 1:100).

Cell culture and luciferase assay

For culturing Caco-2 and HEK293T cells, a complete medium was prepared using serum (Gibco, United States), DEME medium (BasalMedia Technologies, Shanghai, China) and penicillin-streptomycin (BasalMedia Technologies, Shanghai, China). The mouse Fpn luciferase reporter plasmid and the HRE mutant promoters were constructed by cloning the upstream regions as previously described[2] into the pGL4.1-basic vector (Promega, Madison WI) using primers listed in Supplemental Table 4. The luciferase reporters were co-transfected with Hif2α or Hif1α expression vector or empty vector into HEK293T using transfection reagent (Abclonal). Standard dual luciferase assay was used and normalized to a cotransfected control reporter (Promega). HEK293T cells were transiently transfected with wild-type mouse *Fpn* or the mutant luciferase-reporter construct, and co-transfected with empty vector or Hif2α or Hif1α expression plasmids for 40 hours, or treated with FG-4592 for another 3 hours. Unless stated otherwise, the concentration of FG-4592 is 100 μM and the treatment time is 3 hours.

Mouse hypoxia model, Roxadustat (FG-4592) and PT2385 treatment

For hypoxia treatment, 8-week-old male mice were placed in the hypoxia chamber with

the oxygen concentration setting at 10% for 4 weeks. For normoxia treatment, 8-week-old male mice were placed in the normal conditions. For FG-4592 treatment, FG-4592 (S1007, Selleck) was dissolved in sterile saline vehicle, orally gavaged at a concentration of 30 mg/kg per mouse, once a day for 4 weeks, initiated when *Tmprss6*-KO, *Tmprss6*-LKO and littermate mice were of 8 weeks old. For PT2385 treatment, *Tmprss6*-KO mice were orally gavaged at a concentration of 20 mg/kg per mouse, once a day for 3 weeks, initiated when *Tmprss6*-LKO and littermate mice were of 8 weeks old or when mice were exposed in the hypoxia chamber.

5/6 nephrectomy (5/6 Nx)-induced anemia of chronic kidney disease (CKD)

5/6 nephrectomy of male 8-week-old male mice were performed as previously described[3]. Briefly, the 2/3 left kidney of mice was cut at the first week and the whole right kidney was removed two weeks later; Sham operations were performed at the same time points. All mice were raised for 8 weeks to induce anemia of CKD and then fed with vehicle or FG-4592 (30 mg/kg) for 4 weeks.

5-fluorouracil (5-FU)-induced chemotherapy anemia model

Male wild-type mice at 8 weeks of age were randomly divided into 8 groups. All mice were given 150 mg/kg of 5-FU dissolved in sterile vehicle by intraperitoneal injection as previously described[4], except the control group. 5-FU-treated mice were sacrificed at day 7, 14 and 21 after the first injection of 5-FU. Indicated number of mice (n = 3-10 mice/group) were gavaged with FG-4592 (30 mg/kg, once a day) or placed in the hypoxia chamber for one week from day 7 to day 14 after the first injection of 5-FU, while 3 to 10 mice per group were gavaged with FG-4592 (30 mg/kg, once a day) or placed in the hypoxia chamber for two weeks from day 7 to day 21 after the first injection of 5-FU.

Turpentine oil-induced anemia of inflammation

As previously described[5], for the acute anemia of inflammation model 8-week-old male wild-type mice were treated with a single dose of vehicle or turpentine oil (5 ml/kg),

and after two hours, the turpentine oil-treated mice were fed with FG-4592 (30 mg/kg,) or placed in a hypoxia chamber for 14 h, and all the mice were sacrificed 16 h after the first injection of vehicle or turpentine. For the chronic anemia of inflammation model, 8-week-old wild-type mice were continuously treated with turpentine oil (5 ml/kg) for 3 weeks (once a week, four injections in total), then allowed to recover for 2 weeks after the last injection, then gavaged with FG-4592 (30 mg/kg, once a day) or placed in the hypoxia chamber for 3 weeks.

Intestinal crypts extraction, organoid culture, and immunofluorescence staining

Intestinal crypts were extracted from 6-8-week-old male intestinal-specific KO of *Hif1α*, *Hif2*α and control mice. Murine intestinal crypts were isolated following procedures described previously[6]. In brief, the proximal part of the intestine was collected, opened longitudinally and washed with ice-cold PBS for several times to remove luminal content. After washing with ice-cold PBS, the intestine was cut into ~2 mm pieces with scissors. The pieces were transferred to a 50 mL centrifuge tube and further washed with cold PBS (5–10 times) with gentle vortexing. After sedimentation for 1 min, PBS was removed, and intestinal fragments were then incubated and shaken in 25 mL GCDR buffer (Stem Cell, #07174) for 15 min at room temperature, then GCDR buffer was removed, 10 mL of cold PBS (containing 0.1%BSA) was added and the supernatant was passed through a 70 μm strainer. The previous step was repeated a second time. The second collected crypts were centrifuged at the speed of 300 x *g* for 5 mins at 4°C. The pellet was resuspended in 10 mL cold Advanced DMEM/F12 (Stem cell, #36254) and centrifuged at 300 x *g* for 5 mins. Then the resulting pellet was resuspended with IntestiCult™ culture medium (Stem cell) and Matrigel matrix mixture (Corning # 356231) (Volume, 1:1), and was cast into 50 μL dome droplets at the bottom of wells in 24-well plate. FG-4592 was dissolved in IntestiCult™ culture medium (100 μM). Western blotting was performed with anti-Ferroportin (Alpha Diagnostics, 1:1000), anti-HIF1α (Proteintech, 66730-1-Ig, 1:1000) and anti-HIF2α (Abcam, ab109616,1:1000) after FG-4592 or vehicle treatment; and immunofluorescent staining was performed after FG-4592 or vehicle treatment for 3

hours. Organoid cells were fixed in 24-well plates by using 4% paraformaldehyde dissolved in PBS for 20 mins, then washed with PBS. After blocking nonspecific binding sites with 10 % blocking reagent for 20 mins, the cells were incubated with anti-Ferroportin (Alpha Diagnostics, 1:500) at 4 °C for overnight, followed by incubation with the goat anti-rabbit secondary antibody (PV-6001, ORIGEBE) in 37 °C for 30 mins. For tyramide signal amplification (TSA), the cells were incubated with prepared tyramide working solution (Opal 570 reagent #OP-001003 dissolved in Multiplex TSA buffer #322809; 1:200) for 15 mins at room temperature. After heating cells in the 95°C-water bath for 20 mins to wash out the solution, the cells were then incubated with anti-Hif2α antibody (1:100) overnight at 4 °C, followed by incubating the cells with the goat anti-rabbit secondary antibody (PV-6001, ORIGEBE) in 37 °C for 30 mins. For tyramide signal amplification (TSA), the cells were incubated with prepared tyramide working solution (Opal 520 reagent #OP-001001 dissolved in Multiplex TSA buffer #322809; 1:200) for 15 mins at room temperature. Washed out the TSA solution by using PBS for 5min, 3 times followed by adding fluoromount+DAPI solution. The mounted slides were kept at 4°C protected from light[7].

References

1. Yu Y, Jiang L, Wang H, et al. Hepatic transferrin plays a role in systemic iron homeostasis and liver ferroptosis. *Blood*. 2020;136(6):726–739.
2. Taylor M, Qu A, Anderson ER, et al. Hypoxia-inducible factor-2α mediates the adaptive increase of intestinal ferroportin during iron deficiency in mice. *Gastroenterology*. 2011;140(7):2044–2055.
3. Rosendahl A, Kabiri R, Bode M, et al. Adaptive immunity and IL-17A are not involved in the progression of chronic kidney disease after 5/6 nephrectomy in mice. *Br J Pharmacol*. 2019;176(12):2002–2014.
4. Coulon S, Dussiot M, Grapton D, et al. Polymeric IgA1 controls erythroblast proliferation and accelerates erythropoiesis recovery in anemia. *Nat Med*. 2011;17(11):1456–1465.
5. Nicolas G, Chauvet C, Viatte L, et al. The gene encoding the iron regulatory peptide hepcidin is regulated by anemia, hypoxia, and inflammation. *J Clin Invest*. 2002;110(7):1037–1044.
6. Gjorevski N, Sachs N, Manfrin A, et al. Designer matrices for intestinal stem cell and organoid culture. *Nature*. 2016;539(7630):560–564.

7. ELISA: Methods and Protocols. New York, NY: Springer; 2015.

Supplemental Table 1. Sequences of the primers used for genotyping PCR analyses

Primers for genotyping PCR

Gene	Forward primer (5'→3')	Reverse primer (3'→5')
Hif1α	TGTGTCCATGCACCTGTGTG	AGGAGTAAAGAATACGGAAAGACT
Hif2α	TTATAGTGGAATGTGCCCAGGAC	TCCAGAAGCAAAGACCTCCAAG
Tmprss6	TTATGGATCAGTGGAACGTGAG	GCTTACAGAAGAGCAAGCCAAT
Alb-Cre	GCAAACATACGCAAGGGATT	AGGCAAATTTTGGTGTACGG
Gen-Cre	CTGTGGTCTTCCTGCCTTGG	TACCCTGCCCTACACGACTC

Supplemental Table 2. Sequences of the primers used for real-time quantitative PCR analyses

Primers for RT-PCR

Gene	Forward primer (5'→3')	Reverse primer (3'→5')
Mouse *β-actin*	AAATCGTGCGTGACATCAAAGA	GCCATCTCCTGCTCGAAGTC
Mouse *Epo*	GCCTCACTTCACTGCTTCGG	GGAGGCGACATCAATTCCTTC
Mouse *Erfe*	ATGGGGCTGGAGAACAGC	TGGCATTGTCCAAGAAGACA
Mouse *Hamp*	GCACCACCTATCTCCATCAACA	TTCTTCCCCGTGCAAAGG
Mouse *Hif1α*	ATCTCGGCGAAGCAAAGAG	ATGGTGAGCCTCATAACAGAAG
Mouse *Hif2α*	CAGAGCTGAGGAAGGAGAAATC	ATGATGGAGGCTTTGTCCAG
Mouse *Tmprss6*	CTATTGCTTTCCGCAGTGAATC	TGCTGGCAACCAGTTCTT
Mouse *Fpn*	CTACCATTAGAAGGATTGACCAGCTA	ACTGGAGAACCAAATGTCATAATCTG
Mouse *Dmt1(IRE)*	TGTTTGATTGCATTGGGTCTG	CGCTCAGCAGGACTTTCGAG
Mouse *Ncoa4*	TGCCATTGGTCTTCAGGCTCCT	CAGGCATCGCTGAAGAAACTGC
Human *FPN*	CCTTCTCTACCTTGGTCATTCTC	AAAGGAGGCTGTTTCCATAGAG

Supplemental Table 3. Sequences of the primers used for ChIP-qPCR

Primers for ChIP-qPCR

Gene	Forward primer (5'→3')	Reverse primer (3'→5')
Human FPN-1	ATACAACCTGAGGAAGGCAAG	GGTATCACCACAACAGTATCCC
Human FPN-2	TTGCCTGTAACACTGAGAAAGA	AATCCAGGACTGTGCTTCTG
Human FPN-3	CACATCCCAACCGAATCCA	AGACTGACACCCAGTAGTGA
Human FPN-4	CAGGGACACAACTGGGATAAC	CCCTTCACCATCATTCCTTCTC
Human FPN-5	CAGCACCTGACGCTTAGTT	TTCCTGCTCTCCGGGAA

Supplemental Table 4. Sequences of the primers used for luciferase construct

Primers for Luciferase Construct		
Gene	Forward primer (5’→3’)	Reverse primer (3’→5’)
Mouse Fpn	GCGCGCTCGAGTTTCTGTTCTG AG AGGGAA AACATT	GCGCGCAAGCTTTTCAAGAGT GATCCTAAACATTGAA
Fpn-HRE mut-1	CATCACACTTTGAAGAAAGCA TCAACTGAGCTCCTG	CAGGAGCTCAGTTGATGCTTT CTTCAAAGTGTGATG
Fpn-HRE mut-2	TCCTGTTATTGGCACATACCTC CA GGGAATCAGG	CCTGATTCCCTGGAGGTATGT GCCAATAACAGGA

Supplemental Figure 1

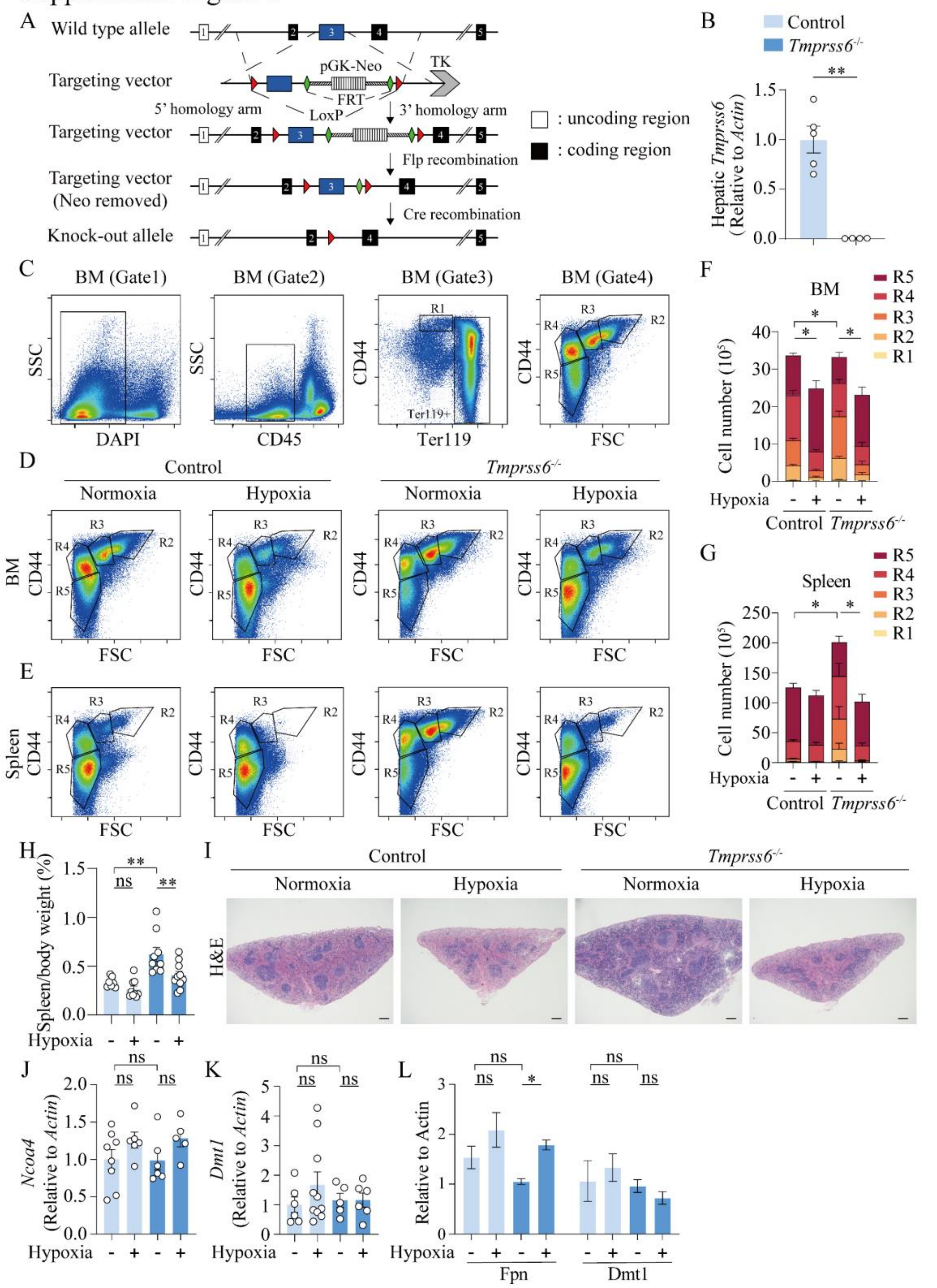

Supplemental Figure 1. Generation and characterization of global *Tmprss6* knockout mice upon hypoxia treatment. (A) A schematic diagram depicting the targeting strategy used to generate the conditional *Tmprss6* knockout allele. **(B)** *Tmprss6* mRNA levels were measured in the livers of male littermate control and *Tmprss6*$^{-/-}$ mice. **(C-G)** Identification of clusters of BM erythroid precursors in WT

littermates **(C)**. (Left) Identification of SSC low DAPI$^-$ cells (Gate 1). Identification of SSC low CD45$^-$ cells (Gate 2). Recognition of erythroid precursors (TER119$^+$ cells and R1, Gate 3) inside the population identified in Gate 2. (Right) Density plot of CD44 vs FSC of cells identified in Gate 3 from TER119$^+$ population showing naturally occurring clusters of erythroid precursors at progressive maturation stages (R2-R5). **(D)** Density plots of R2-R5 cells in BM of normoxia- or hypoxia- treated control and *Tmprss6*$^{-/-}$ mice. **(E)** Density plots showing representative distribution of splenic R2-R5 cells of normoxia- or hypoxia- treated control and *Tmprss6*$^{-/-}$mice. **(F-G)** Quantification of BM **(F)** and spleen **(G)** erythroid precursors R1-R5. Quantification was performed on samples from at least 4-5 mice for every condition. In the bone marrow, R2-R3 ($p<0.05$): normoxia-treated *Tmprss6*$^{-/-}$ mice *vs.* normoxia-treated control mice; R4-R5 ($p<0.05$): normoxia-treated *Tmprss6*$^{-/-}$ mice *vs.* normoxia-treated control mice; R2-R4 ($p<0.05$): hypoxia-treated control mice *vs.* normoxia-treated control mice; R5 ($p<0.05$): hypoxia-treated control mice *vs.* normoxia-treated control mice; R2-R4 ($p<0.05$): hypoxia-treated *Tmprss6*$^{-/-}$ mice *vs.* normoxia-treated *Tmprss6*$^{-/-}$ mice; R5 ($p<0.05$): hypoxia-treated *Tmprss6*$^{-/-}$ mice *vs.* normoxia-treated *Tmprss6*$^{-/-}$ mice. In the spleen, R2-R4 ($p<0.05$): normoxia-treated *Tmprss6*$^{-/-}$ mice *vs.* normoxia-treated control mice; R5 ($p<0.05$): normoxia-treated *Tmprss6*$^{-/-}$ mice *vs.* normoxia-treated control mice. R2-R4 ($p<0.05$): hypoxia-treated *Tmprss6*$^{-/-}$ mice *vs.* normoxia-treated *Tmprss6*$^{-/-}$ mice; R5 ($p<0.05$): hypoxia-treated *Tmprss6*$^{-/-}$ mice *vs.* normoxia-treated *Tmprss6*$^{-/-}$ mice. R1: proerythroblasts; R2: basophilic erythroblasts; R3: polychromatophilic erythroblasts; R4: orthochromatophilic erythroblasts; R5: mature erythrocytes. **(H)** Spleen mass divided by body weight was calculated as Spleen/body weight (%) in normoxia- or hypoxia- treated control and *Tmprss6*$^{-/-}$ mice. **(I)** Representative images of spleen H&E staining in normoxia- or hypoxia- treated control and *Tmprss6*$^{-/-}$ mice; the scale bars represent 200 μm. **(J-L)** *Ncoa4* **(J)** and *Dmt1* **(K)** mRNA levels in the duodenum, quantification of blots for Fpn and Dmt1 **(L)** were detected in normoxia- or hypoxia-treated control and *Tmprss6* $^{-/-}$mice. (*n*=4-10 mice/group). Two-way ANOVA with Tukey's post hoc test (for multi-group comparisons).

Supplemental Figure 2

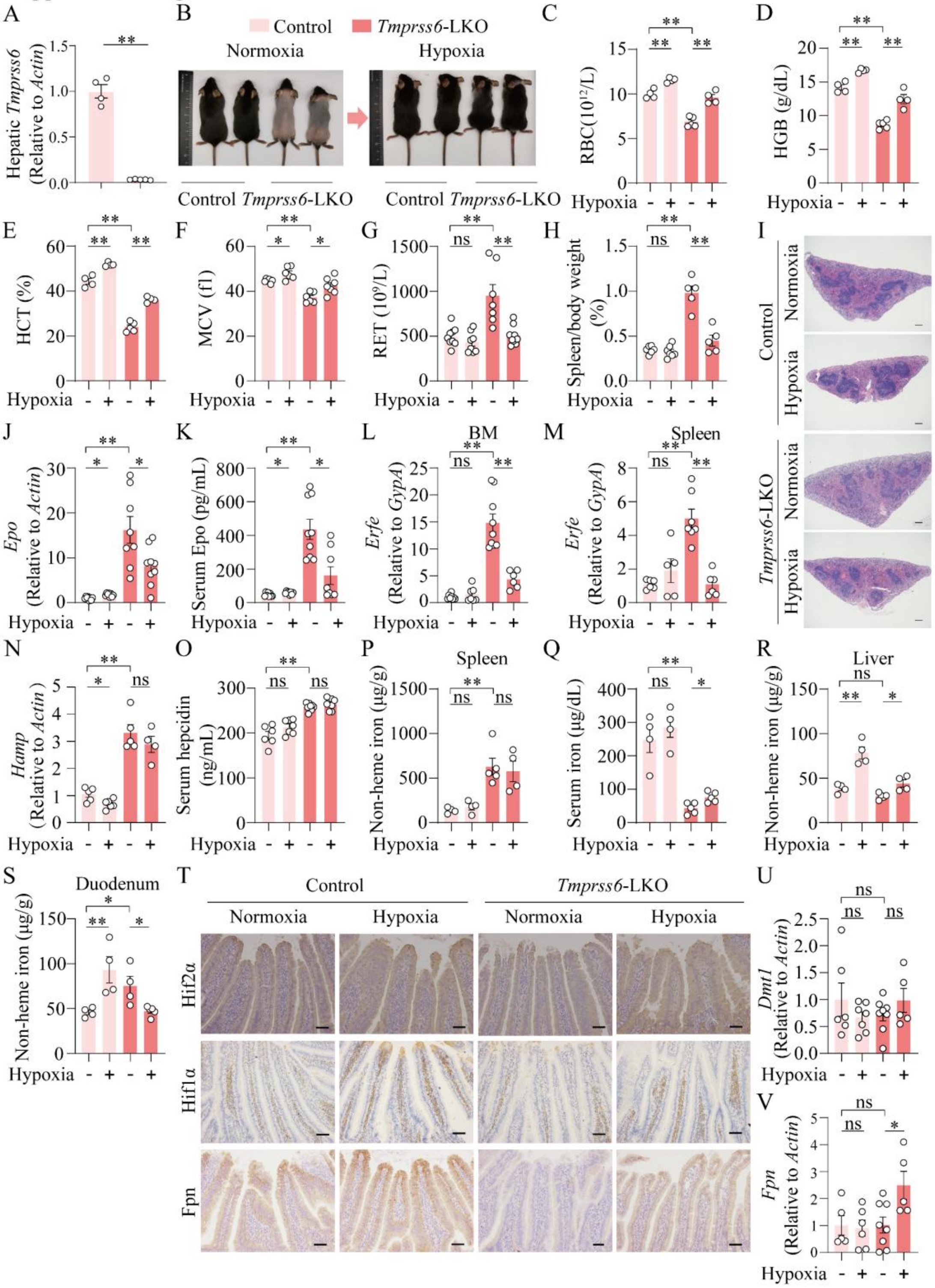

Supplemental Figure 2. Hepatic *Tmprss6* knockout mice display improved phenotypes of IRIDA upon hypoxia exposure for 4 weeks. (A) Hepatic *Tmprss6* mRNA levels were measured in control (*Tmprss6*$^{flox/flox}$) and *Tmprss6*-LKO mice. ***p*<0.01, Student's *t*-test. **(B)** Representative images of 8-week-old control and liver-specific *Tmprss6* knockout (*Tmprss6*-LKO) mice before and after placement in a

hypoxia chamber (O_2:10%) for 4 weeks. **(C-G)** RBC **(C)**, HGB **(D)**, HCT **(E)**, MCV **(F)** and RET **(G)** values in normoxia- and hypoxia-exposed control and *Tmprss6*-LKO mice. **(H)** Spleen mass divided by body weight was calculated as Spleen/body weight (%) in normoxia- or hypoxia- treated control and *Tmprss6*-LKO mice. **(I)** Representative images of spleen H&E staining in normoxia- or hypoxia- treated control and *Tmprss6*-LKO mice; the scale bars represent 200 μm. **(J-O)** *Epo* mRNA levels in the kidney **(J)** and Epo protein levels in the serum **(K)**, *Erfe* mRNA levels normalized relative to the erythroid marker *GypA* in the bone marrow **(L)** and spleen **(M)**, and *Hamp* mRNA levels in the liver **(N)** and hepcidin protein levels in the serum **(O)** were detected in normoxia- and hypoxia-exposed *Tmprss6*-LKO and control mice. **(P-S)** Splenic non-heme iron **(P)**, serum iron **(Q)** and hepatic **(R)** and duodenal non-heme iron concentration **(S)** were detected in normoxia- and hypoxia- exposed control and *Tmprss6*-LKO mice. **(T)** Hif2α, Hif1α and Fpn IHC staining of duodenum sections obtained from normoxia- and hypoxia- treated control and *Tmprss6*-LKO mice; the scale bars represent 100 μm. **(U-V)** Duodenal *Dmt1* **(U)** and *Fpn* **(V)** mRNA levels were tested in normoxia- and hypoxia-exposed *Tmprss6*-LKO and control mice. (*n*=4-9 mice/group). Two-way ANOVA with Tukey's post hoc test (for multi-group comparisons).

Supplemental Figure 3

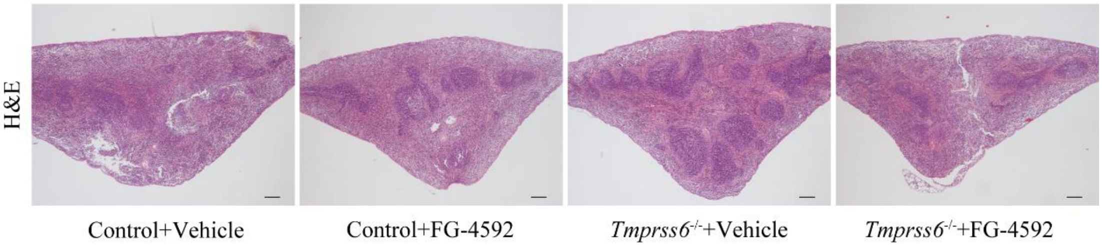

Supplemental Figure 3. Characterization of global *Tmprss6* knockout mice display improved phenotypes of IRIDA upon FG-4592 for 4 weeks. Representative images of spleen H&E staining in vehicle- or FG-4592- treated control and *Tmprss6*$^{-/-}$ mice; the scale bars represent 200 μm.

Supplemental Figure 4

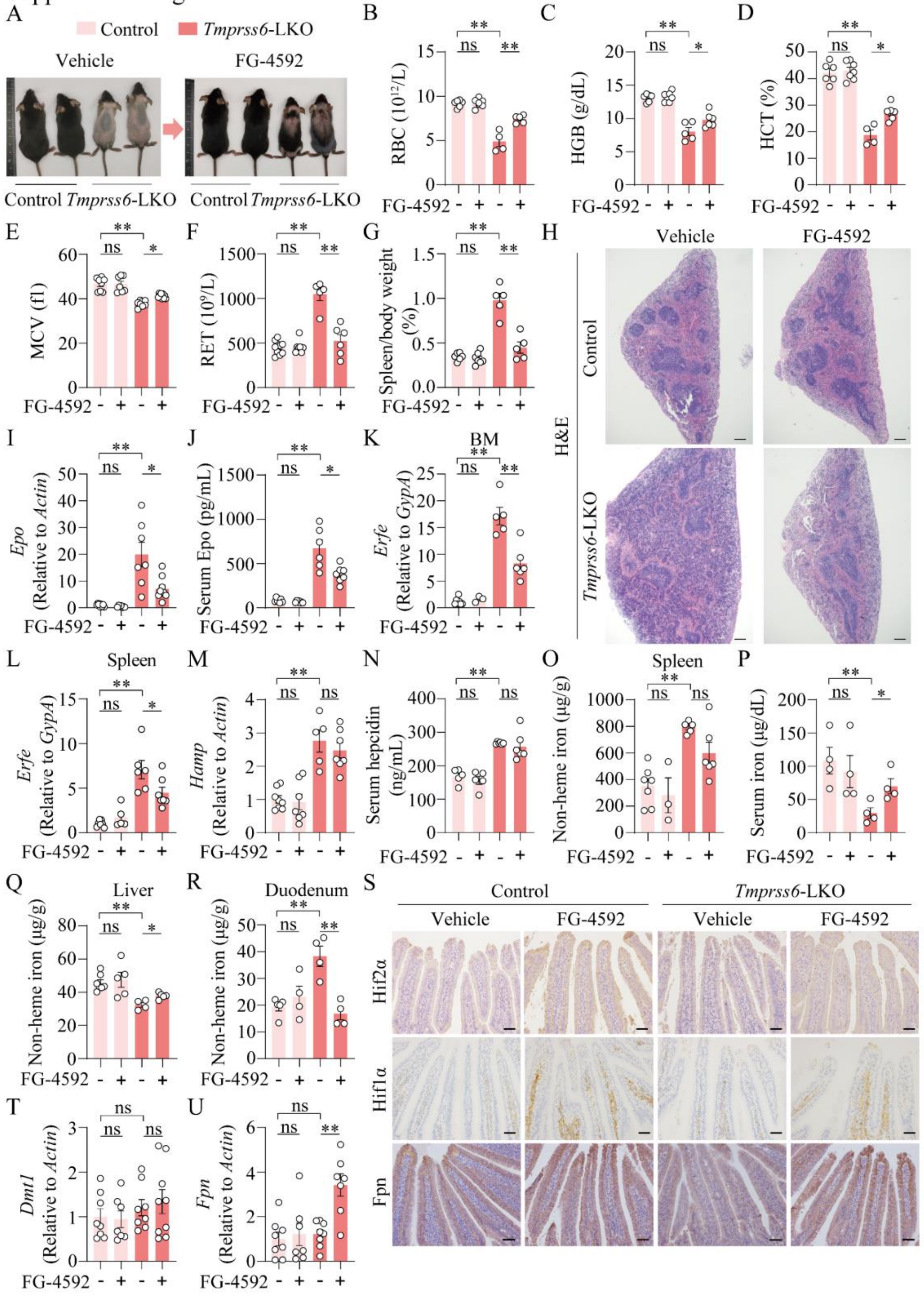

Supplemental Figure 4. Hepatic *Tmprss6* knockout mice display improved phenotypes of IRIDA upon FG-4592 treatment for 4 weeks. (A) Representative images of 8-week-old control and *Tmprss6*-LKO mice before and after gavage with FG-4592 for 4 weeks (once a day, 30 mg/kg). **(B-F)** RBC **(B)** HGB **(C)** HCT **(D)**MCV **(E)** and RET **(F)** levels in saline vehicle- and FG-4592-treated control and *Tmprss6*-

LKO mice. **(G)** Spleen mass divided by body weight was calculated as Spleen/body weight (%) in vehicle- and FG-4592- treated control and *Tmprss6*-LKO mice. **(H)** Representative images of H&E staining for spleen sections in vehicle- and FG-4592- treated control and *Tmprss6*-LKO mice, the scale bars represent 200 μm. **(I-N)** *Epo* mRNA levels in kidney **(I)**, Serum Epo levels **(J)**, *Erfe* mRNA levels normalized to *GypA* in the bone marrow **(K)** and spleen **(L)**, *Hamp* mRNA levels in the liver **(M)** and serum hepcidin levels **(N)** were detected in vehicle- and FG-4592- treated control and *Tmprss6*-LKO mice. **(O-R)** Splenic iron levels **(O)**, serum iron **(P)** and hepatic non-heme iron concentration **(Q)** and duodenal iron content **(R)** were detected in vehicle- and FG-4592- treated control and *Tmprss6*-LKO mice. **(S)** Representative images of immunohistochemistry for Hif2α, Hif1α and Fpn IHC staining of duodenum sections obtained from vehicle- and FG-4592- treated control and *Tmprss6*-LKO mice; the scale bars represent 100 μm. **(T-U)** Duodenal *Dmt1* **(T)** and *Fpn* **(U)** mRNA levels were tested in vehicle- and FG-4592- treated *Tmprss6*-LKO and control mice. (*n*=3-10 mice/group). Two-way ANOVA with Tukey's post hoc test (for multi-group comparisons).

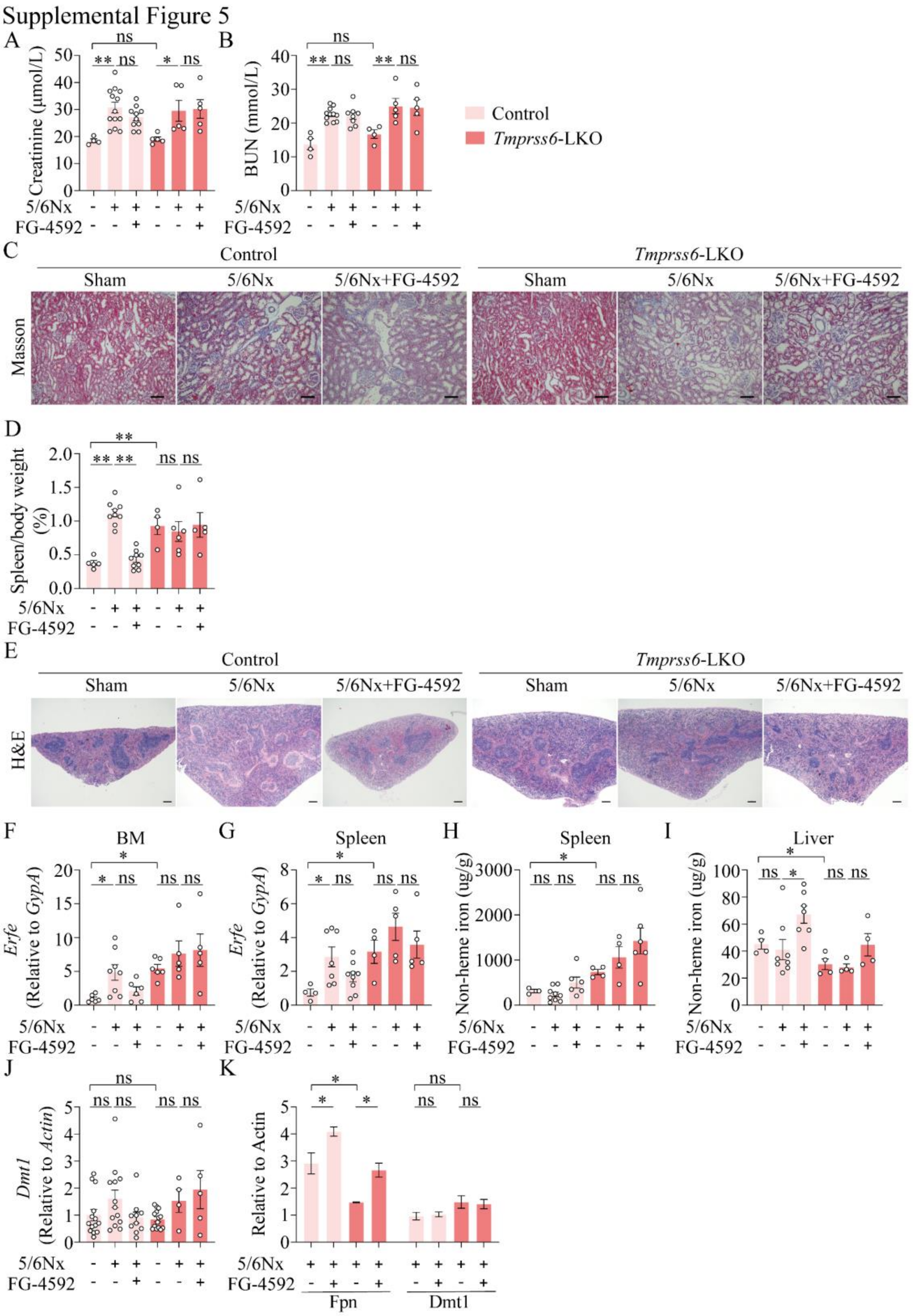

Supplemental Figure 5. Mice operated with 5/6 nephrectomy showed altered Masson trichrome staining in the kidney. (A-B) Serum creatinine **(A)** and BUN **(B)** were tested in vehicle- and FG-4592-treated control and *Tmprss6*-LKO mice with 5/6Nx. **(C)** Representative images of the kidney sections stained with Masson's trichrome, the scale bars represent 100 μm. **(D)** Spleen mass divided by body weight

was calculated as Spleen/body weight (%) in vehicle- and FG-4592-treated control and *Tmprss6*-LKO mice with 5/6Nx. **(E)** Representative images of H&E staining for spleen sections in vehicle- and FG-4592-treated control and *Tmprss6*-LKO mice with 5/6Nx, the scale bars represent 200 μm. **(F-G)** *Erfe* mRNA normalized to *GypA* in the bone marrow **(F)** and spleen **(G)** were detected in vehicle- and FG-4592-treated control and *Tmprss6*-LKO mice with 5/6Nx. **(H-I)** Splenic non-heme iron **(H)** and hepatic non-heme iron levels **(I)** were detected in vehicle- and FG-4592-treated control and *Tmprss6*-LKO mice with 5/6Nx. **(J)** Duodenal *Dmt1* mRNA levels were tested in vehicle- and FG-4592-treated control and *Tmprss6*-LKO mice with 5/6Nx. **(K)** The Fpn and Dmt1 protein levels were quantified in vehicle- and FG-4592-treated control and *Tmprss6*-LKO mice with 5/6Nx. (*n*=4-13 mice/group). Two-way ANOVA with Tukey's post hoc test (for multi-group comparisons).

Supplemental Figure 6

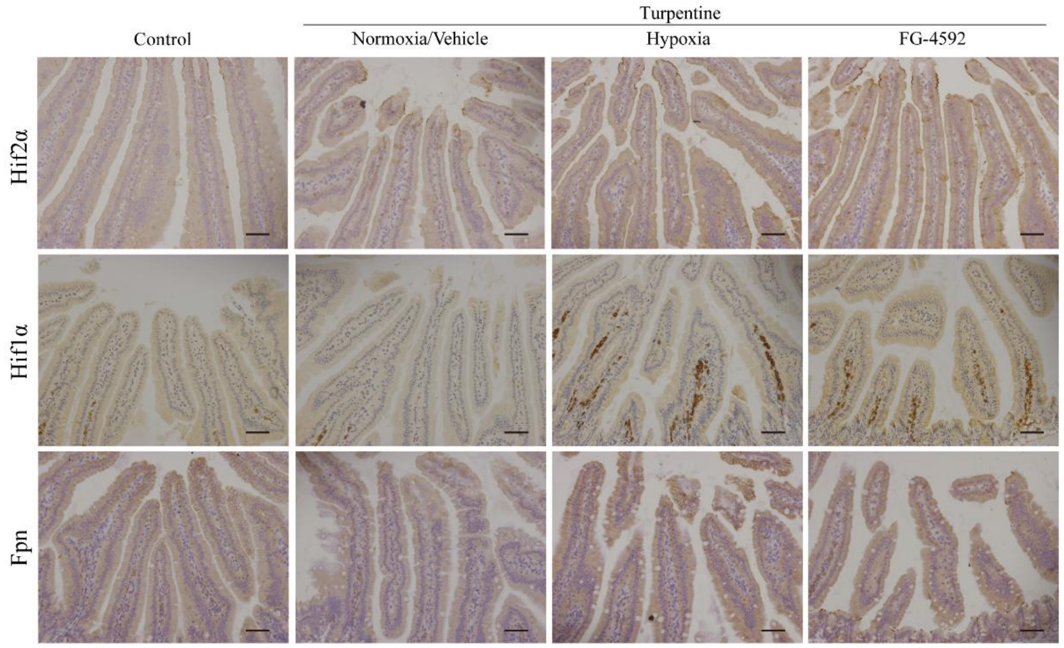

Supplemental Figure 6. The effect of FG-4592 or hypoxia on acute Turpentine oil treated inflammation. In the acute Turpentine oil treated model, duodenum Hif2α, Hif1α and Fpn staining were performed in the indicated four groups; the scale bars represent 100 μm.

Supplemental Figure 7

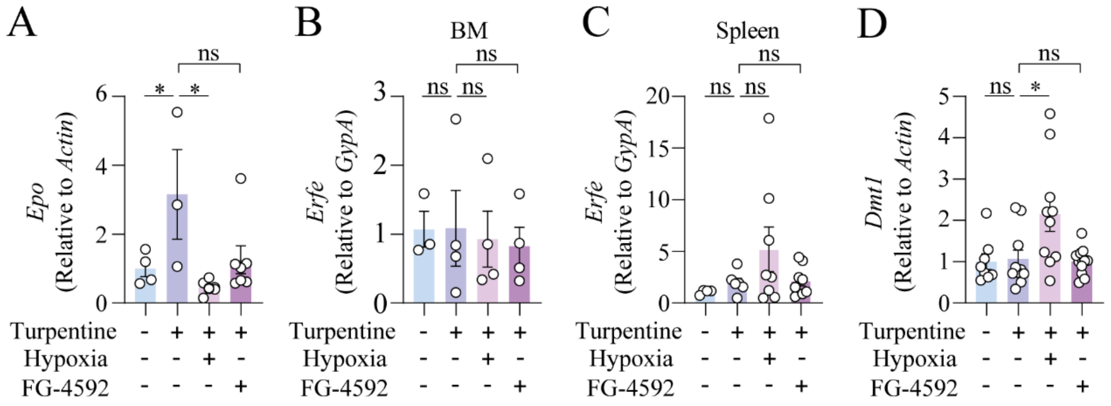

Supplemental Figure 7. Characterization of mice fed with FG-4592 or treated with hypoxia in chronic anemia of inflammation. (A-D) In the chronic Turpentine oil treated model, renal *Epo* **(A)**, and *Erfe* levels normalized to *GypA* in the bone marrow **(B)** and spleen **(C)**, and duodenal *Dmt1* levels **(D)** were detected in vehicle-treated, turpentine oil-treated, hypoxia and Turpentine oil-treated, and FG-4592 fed-and Turpentine oil-treated wildtype mice. (*n*=3-12 mice/group). One-way ANOVA with Tukey's post hoc test (for multi-group comparisons).

Supplemental Figure 8

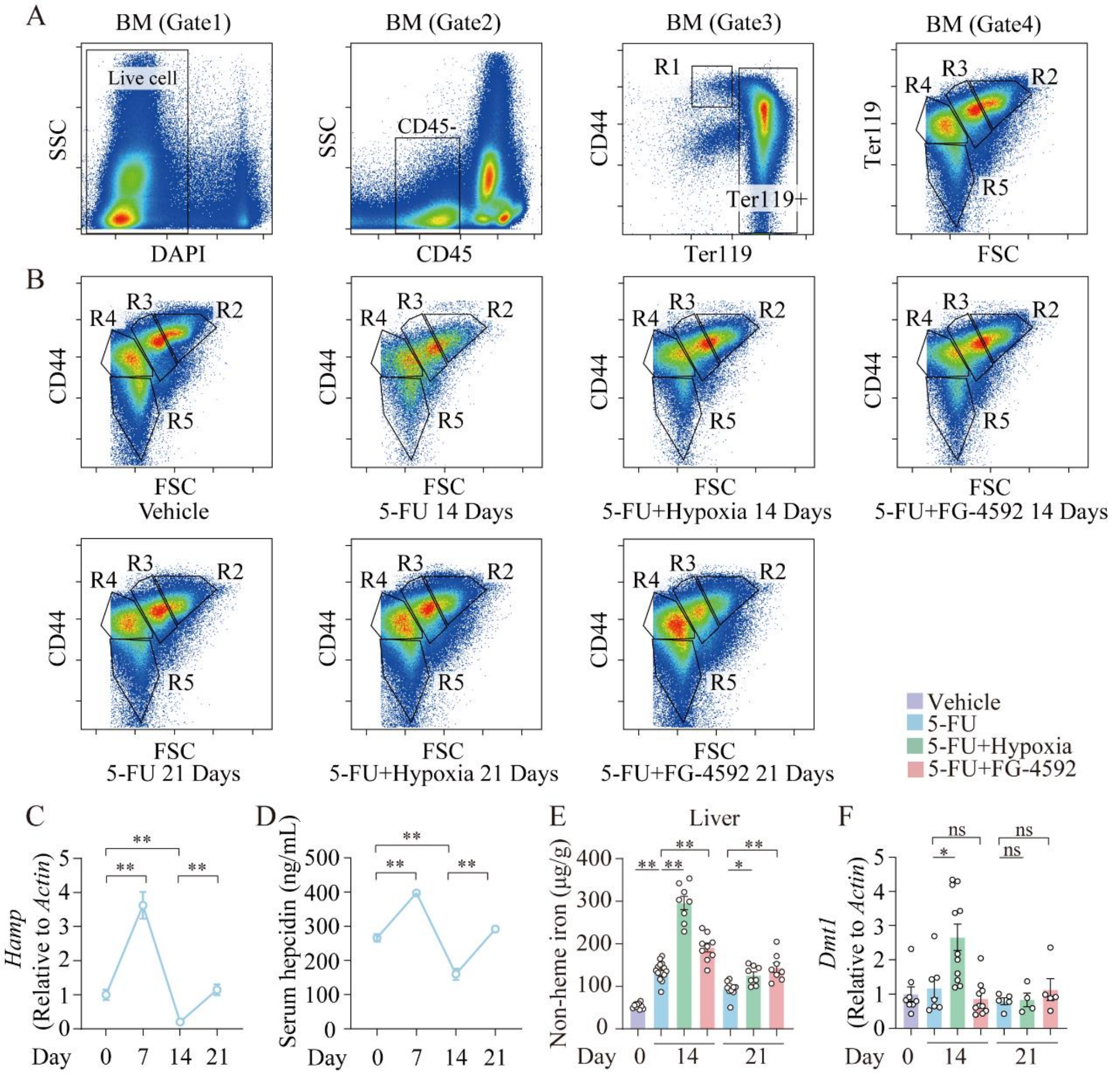

Supplemental Figure 8. Hepatic *Hamp* and serum Hepcidin levels in 5-fluorouracil induced chemotherapy-related anemia. (A-B) Identification of clusters of BM erythroid precursors in WT littermates **(A)**. (Left) Identification of SSC low DAPI$^-$ cells (Gate 1). Identification of SSC low CD45$^-$ cells (Gate 2). Recognition of erythroid precursors (TER119$^+$ cells and R1, Gate 3) inside the population identified in Gate 2. (Right) Density plot of CD44 vs FSC of cells identified in Gate 3 showing naturally occurring clusters of erythroid precursors at progressive maturation stages (R2-R5). **(B)** Density plots of R2-R5 cells in BM at 0-, 14- and 21-days post 5-FU injections, and with/without hypoxia or FG-4592 treatment. **(C-D)** Real-time qPCR for hepatic *Hamp* **(C)** and ELISA for serum Hepcidin **(D)** at 0-, 7-, 14- and 21-days post 5-FU injections. Mice were sacrificed at day 0, 7, 14 and 21 after the first injection of 5-FU, respectively **(E-F)** Hepatic iron **(E)** and duodenal *Dmt1* levels **(F)** were detected at day 0, 14 and 21 after the first injection of 5-FU, with/without hypoxia or FG-4592 treatment. (*n*=4-15 mice/group). One-way ANOVA with Tukey's post hoc test (for multi-group comparisons).

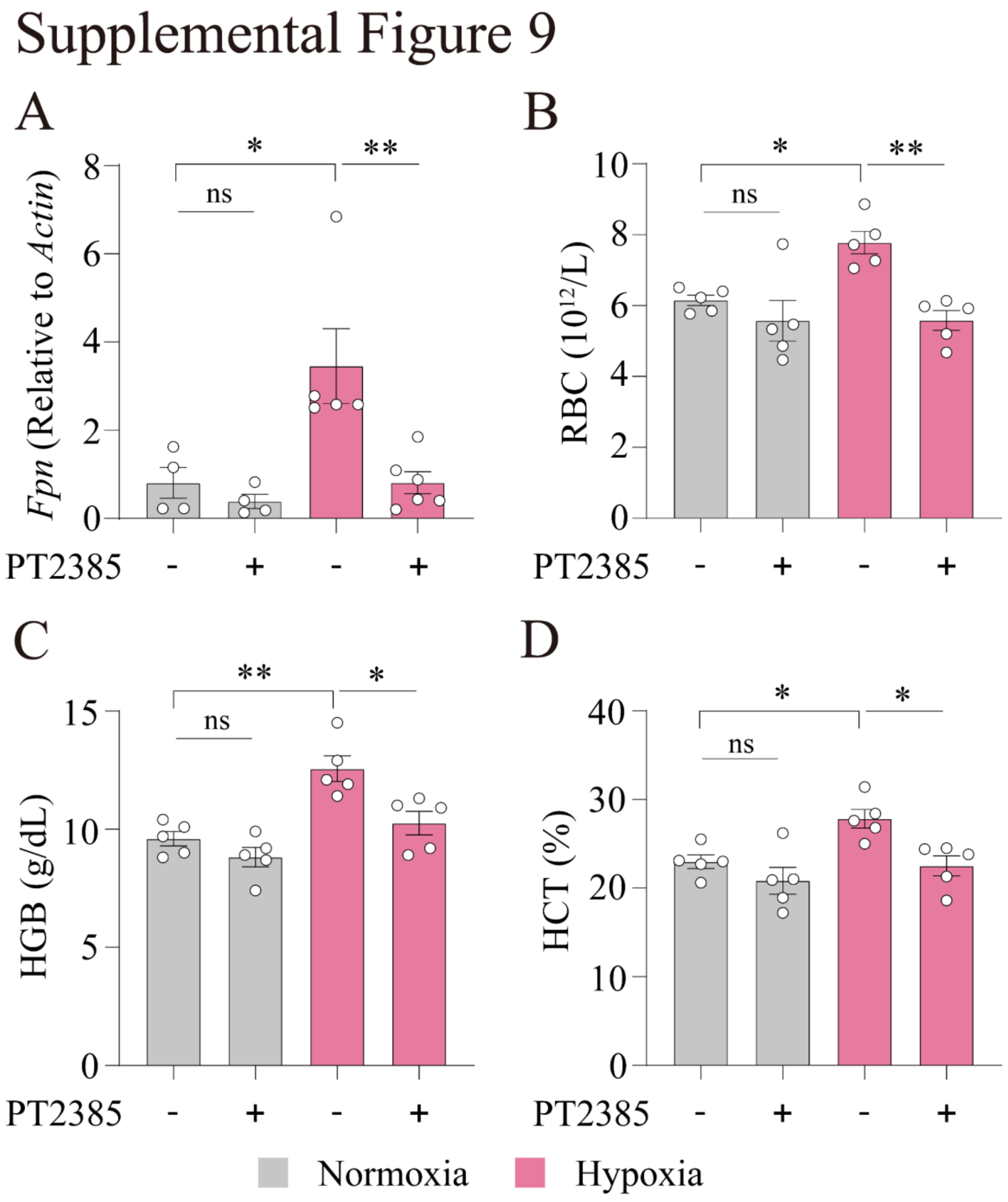

Supplemental Figure 9. PT2385 treatment blocked anemic recovery by hypoxia treatment in *Tmprss6*$^{-/-}$ mice. (A) Real-time PCR for duodenum *Fpn* in normoxia- or hypoxia- exposed *Tmprss6*$^{-/-}$ mice with or without PT2385 treatment. **(B-D)** RBC **(B)**, HGB **(C)** and HCT **(D)** values in normoxia- and hypoxia-exposed *Tmprss6*$^{-/-}$ mice with or without PT2385 treatment. (*n*=5-6 mice/group). Two-way ANOVA with Tukey's post hoc test (for multi-group comparisons).

Supplemental Figure 10

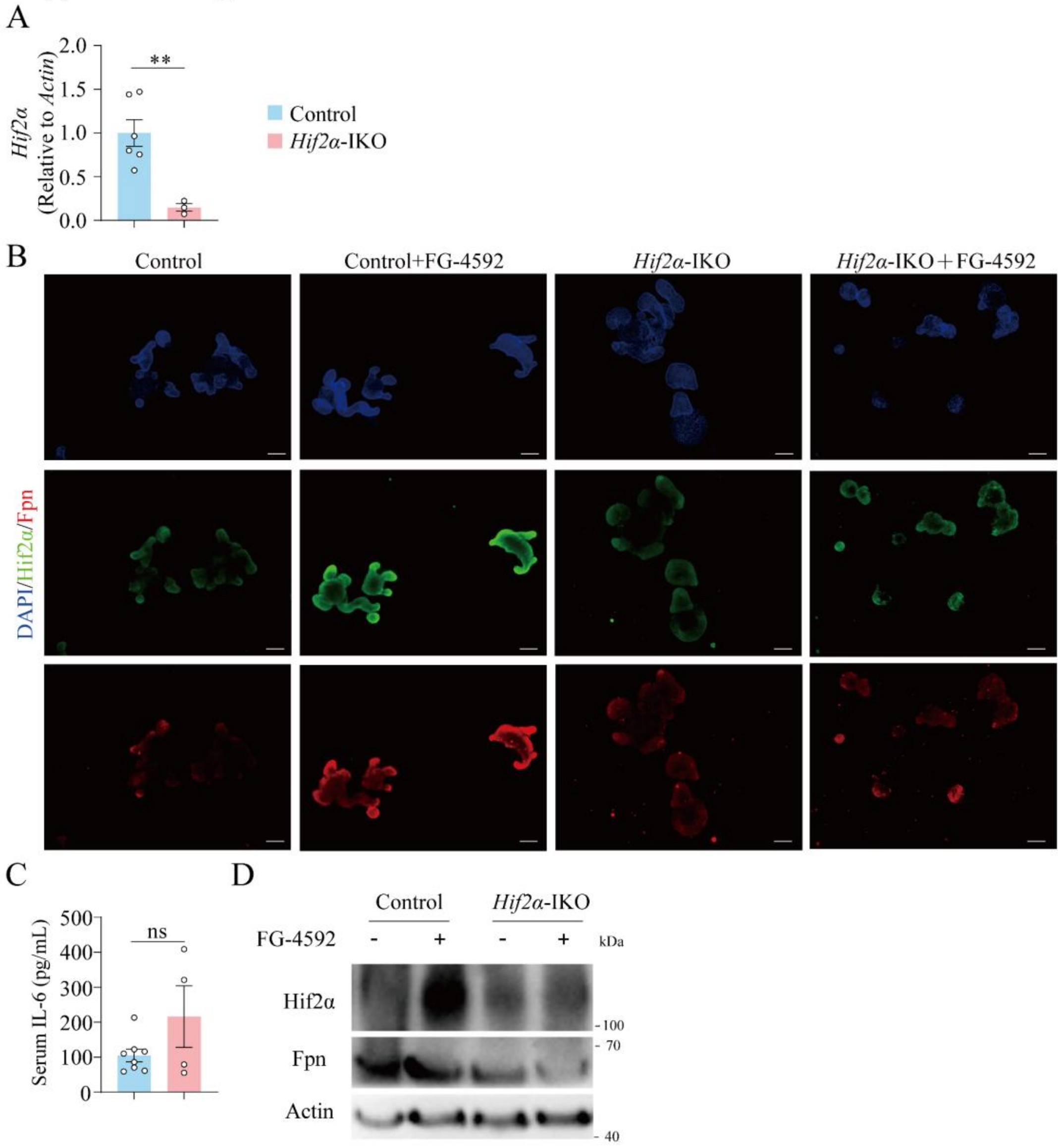

Supplemental Figure 10. Intestine *Hif2α* is required for recovery from 5-fluorouracil induced chemotherapy-related anemia. (A) Real-time PCR for duodenum *Hif2α* in Intestine-specific *Hif2α* knockout (*Hif2α*-IKO) and control mice. **(B)** Representative images of immunofluorescent staining for Hif2α and Fpn from the organoids described in Figure 7A and treated with 100 μM FG-4592 or vehicle for 3 hours. **(C)** Serum IL-6 levels were measured in littermate control and *Hif2α*-IKO mice at 21 days after 5-FU injection. (*n*=3-8 mice/group) $^{**}p<0.01$, ns= not significant (Student's *t*-test). **(D)** Representative results of western blot for Hif2α and Fpn protein levels from control and *Hif2α*-IKO mice at 21 days after 5-FU injection with or without FG-4592 treatment.

Supplemental Figure 11

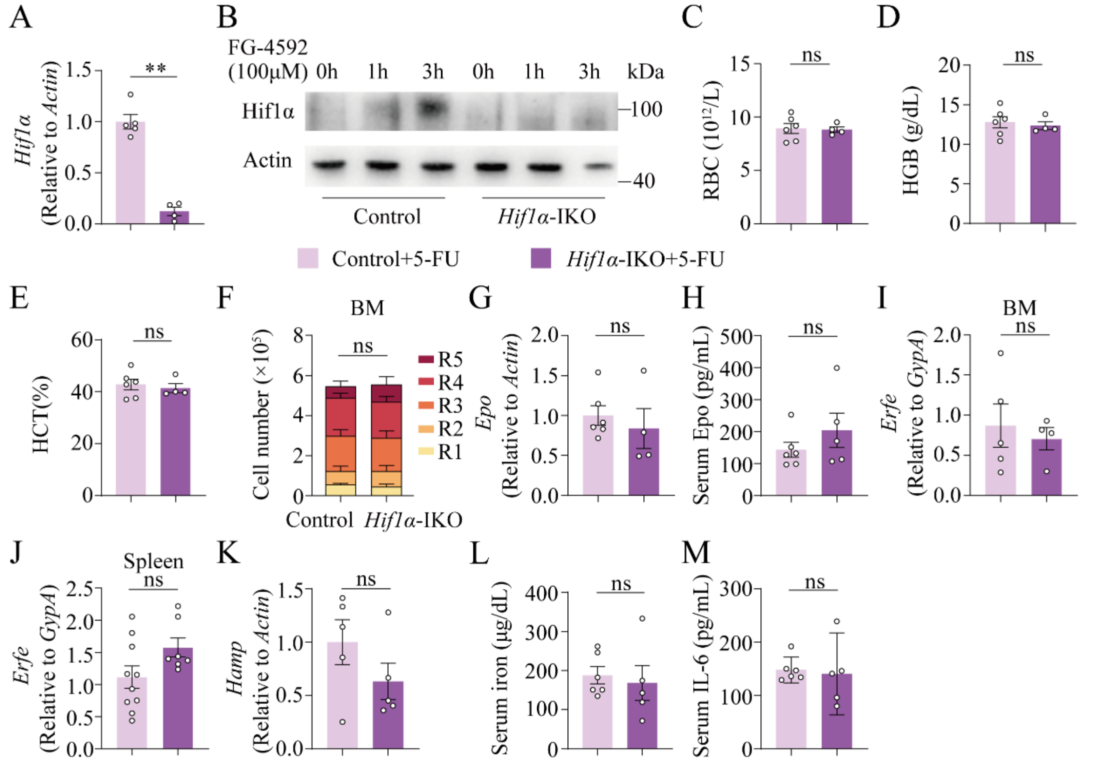

Supplemental Figure 11. Intestine *Hif1α* is not required for recovery from 5-fluorouracil induced chemotherapy-related anemia. (A) Real-time qPCR for duodenum *Hif1α* in littermate control and Intestine-specific *Hif1α* knockout (*Hif1α*-IKO) mice (*n*=3-7 mice/group) at day 21 after 5-FU injection. **(B)** Control and intestine-specific *Hif1α* knockout mice (*Hif1α*-IKO) were used to establish *in vitro* intestinal organoids, which were treated with 100 μM FG-4592 for 1-3 hours for the protein testing. **(C-E)** RBC **(C)**, HGB **(D)**, and HCT values **(E)** were measured in littermate control and Intestine-specific *Hif1α* knockout (*Hif1α*-IKO) mice at day 21 after 5-FU injection. **(F)** Flow cytometry analysis of erythroid cell populations (R1-R5) in bone marrow stained with anti-CD44 and anti-TER-119 in control and Intestine-specific *Hif1α* knockout mice at day 21 of 5-FU injection (*n*=3-4 mice/group). No statistical significance was found in respective R1-R5 counts between control and *Hif1α*-IKO mice after 5-FU injection. **(G-K)** Renal *Epo* **(G)**, serum Epo **(H)**, *Erfe* normalized to *GypA* in the bone marrow **(I)** and spleen **(J)**, hepatic *Hamp* **(K)**, serum iron **(L)** and IL-6 levels **(M)** were measured in littermate control and *Hif1α*-IKO mice at 21 days after 5-FU injection. (*n*=4-10 mice/group). ***P*<0.01, ns= not significant (Student's *t*-test).
